# Supplementary material for: Neuron type‐specific increase in lamin B1 contributes to nuclear dysfunction in Huntington’s disease
Source: EMBO Mol Med. 2020 Dec 28;13(2):e12105. doi: 10.15252/emmm.202012105 (PMC7863407; doi:10.15252/emmm.202012105)
Supplement: Supplementary file 1 — Appendix [file EMMM-13-e12105-s001.pdf]

APPENDIX

Neuron type-specific increase in lamin B1 contributes to nuclear dysfunction in Huntington's disease

Rafael Alcalá-Vida[1,2,3,10,§], Marta Garcia-Forn[1,2,3,11,§], Carla Castany-Pladevall[1,2,3], Jordi Creus-Muncunill[1,2,3], Yoko Ito[4], Enrique Blanco[5], Arantxa Golbano[1,2,3], Killian Crespí-Vázquez[1,2,3], Aled Parry[6], Guy Slater[4], Shamith Samarajiwa[7], Sandra Peiró[8], Luciano Di Croce[5,9], Masashi Narita[4], Esther Pérez-Navarro[1,2,3,*]

[1]Departament de Biomedicina, Facultat de Medicina i Ciències de la Salut, Institut de Neurociències, Universitat de Barcelona, 08036 Barcelona, Catalonia.

[2]Institut d'Investigacions Biomèdiques August Pi i Sunyer (IDIBAPS), Barcelona, Catalonia.

[3]Centro de Investigación Biomédica en Red sobre Enfermedades Neurodegenerativas (CIBERNED), Spain.

[4]Cancer Research UK Cambridge Institute, University of Cambridge, Cambridge, UK

[5]Centre for Genomic Regulation (CRG), The Barcelona Institute of Science and Technology, Dr. Aiguader 88, Barcelona, Spain.

[6]Epigenetics Programme, The Babraham Institute, Cambridge, UK

[7]MRC Cancer Unit, Hutchison/MRC Research Centre, University of Cambridge, Cambridge Biomedical Campus, Cambridge, CB2 0XZ, UK.

[8]Vall d'Hebron Institute of Oncology, 08035, Barcelona, Spain.

[9]ICREA, Pg. Lluis Companys 23, Barcelona, Spain.

[10]Present address: Laboratory of Cognitive and Adaptive Neuroscience, UMR 7364 (CNRS/Strasbourg University), Strasbourg, France.

[11]Present address: Seaver Autism Center for Research and Treatment, Icahn School of Medicine at Mount Sinai, New York, NY 10029, USA.

[§]Equal contribution

*Correspondence to: Esther Pérez-Navarro

Departament de Biomedicina, Facultat de Medicina i Ciències de la Salut, Institut de Neurociències, Universitat de Barcelona, 08036 Barcelona, Catalonia.

E-mail: estherperez@ub.edu

CONTENT

Appendix Figure S1. Lamin B1 levels do not correlate with the age of the individuals

Appendix Figure S2. Lamin B1 protein levels do not correlate with the number of CAG repeats in HD patients

Appendix Figure S3. Lamin B1 levels are not altered in R6/1 mouse striatal oligodendrocytes

Appendix Figure S4. Lamin B1 localization in 12-week-old wild-type and R6/1 mouse hippocampus

Appendix Figure S5. Alterations in lamin B1 in the nuclei from R6/1 mouse striatum and hippocampus occur independently of the presence of mHtt inclusions

Appendix Figure S6. Nuclear markers for striatal MSNs and hippocampal CA1 and DG neurons

Appendix Figure S7. Lamin B1 levels and nuclear morphology are not altered in R6/1 striatal cells nuclei

Appendix Figure S8. Analysis of MSNs and glial cells in the putamen of HD patients by FANSI

Appendix Figure S9. Dextran freely penetrates in striatal and hippocampal neurons nuclei

Appendix Figure S10. Lamin B1 Chip-seq and nuclear sub-fractionation additional data

Appendix Figure S11. ATAC-seq and RNA-seq additional data

Appendix Figure S12. Effects of betulinic acid in the striatum and hippocampus at the molecular level

Appendix Figure S13. Selection of nuclei from neuronal populations

Appendix Figure S14. Representation of FRAP recovery curve with the parameters analyzed

Appendix Table S1. Details on human *post-mortem* brain samples

Appendix Table S2. Details on antibodies used in the study

Appendix Table S3. Summary of statistical tests and p values for Main Figures

Appendix Table S4. Summary of statistical tests and p values for EV Figures

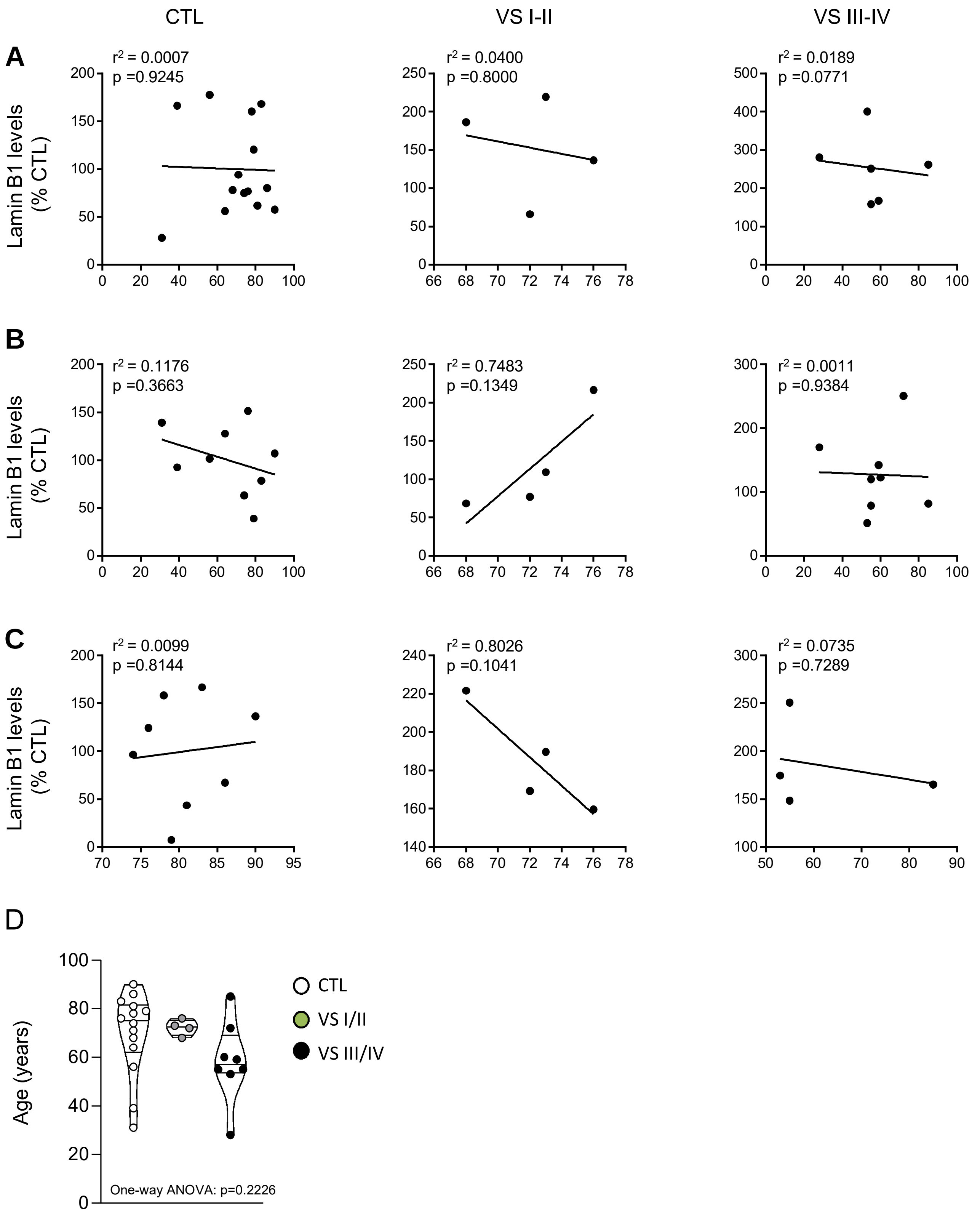

Appendix Figure S1. Lamin B1 protein levels do not correlate with the age of the individuals.

A-C Correlation between lamin B1 levels and individuals' age in the putamen, hippocampus and cortex, respectively, of control individuals (CTL) and HD patients at different VS stages as determined by simple linear regression. In A, CTL *N* = 14, VS I-II *N* = 4 and VS III-IV *N* = 6. In B, CTL *N* = 9, VS I-II *N* = 4 and VS III-IV *N* = 8. In C, CTL *N* = 8, VS I-II *N* = 4 and VS III-IV *N* = 4.

D Violin plot showing the distribution of individuals' ages (years) within each group. Each point corresponds to the value from an individual sample. CTL *N* = 14; VS I/II *N* = 4; VS III-IV *N* = 8.

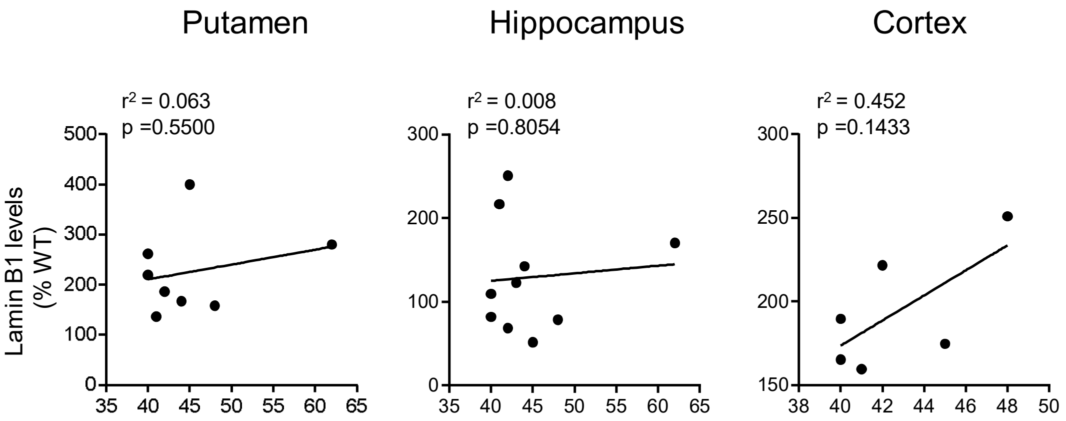

Appendix Figure S2. Lamin B1 protein levels do not correlate with the number of CAG repeats in HD patients.

Correlation between lamin B1 levels and CAG repeats in the putamen, hippocampus and cortex of HD patients at different VS stages as determined by simple linear regression. Each point corresponds to the value from an individual simple. Putamen N = 8; Hippocampus N = 10 and Cortex N = 6.

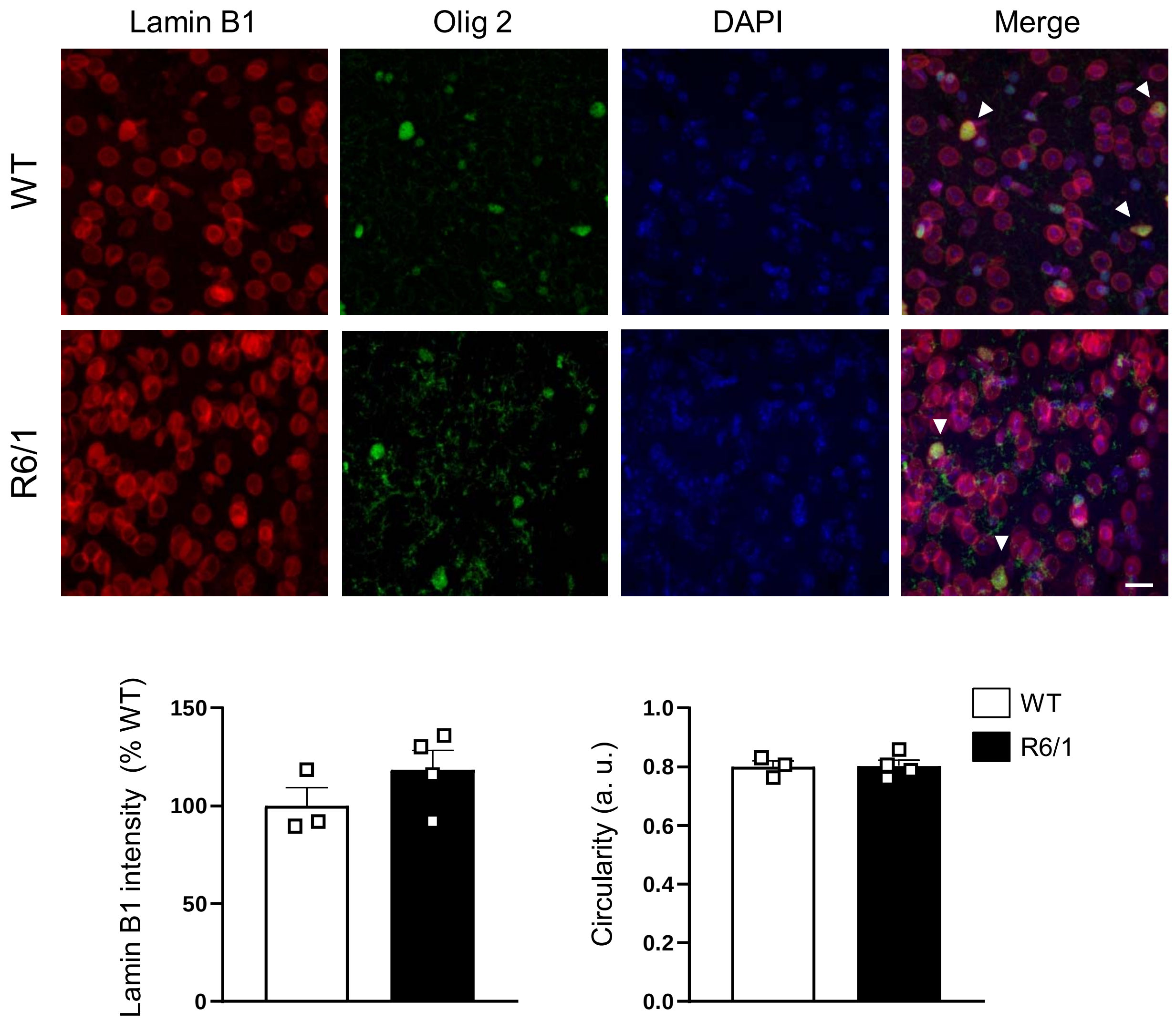

Appendix Figure S3. Lamin B1 levels are not altered in R6/1 mouse striatal oligodendrocytes.

Lamin B1 intensity and nuclear circularity were analyzed by immunohistochemistry in striatal oligodendrocytes from wild-type (WT) and R6/1 mice at 30 weeks of age. Antibody against lamin B1 (red) was combined with anti-Olig2 antibody (green) to label oligodendrocytes and DAPI-Fluoromount G (blue) were used to label nuclei. Representative images are shown. Each point corresponds to the value from an individual sample (WT *N* = 3; R6/1 *N* = 4). Bars represent the mean ± S.E.M. White arrowheads indicate oligodendrocytes. Scale bar 14 µm.

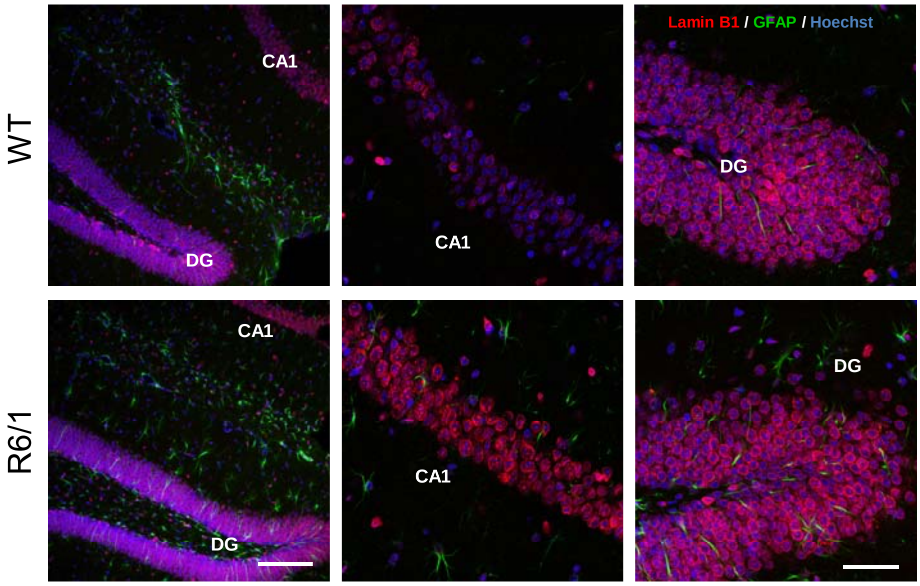

Appendix Figure S4. Lamin B1 localization in 12-week-old wild-type and R6/1 mouse hippocampus

Hippocampal sections were labelled with anti-lamin B1 antibody (red), anti-GFAP antibody (green) and nuclei were labelled with DAPI-Fluoromount G (blue). Representative images of maximal Z-projection are shown. Scale bars 150 µm and 20 µm for low and high magnification, respectively. WT: wild-type mouse.

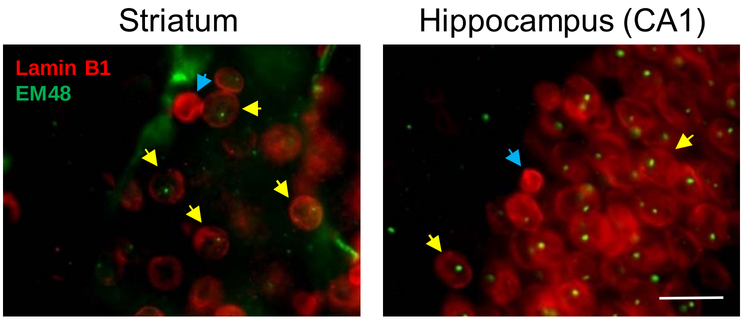

Appendix Figure S5. Alterations in lamin B1 in nuclei from R6/1 mouse striatum and hippocampus occur independently of the presence of mHtt inclusions.

Representative images showing lamin B1-positive nuclei (red) and mHtt inclusions labeled with the EM48 antibody (green) in the striatum and hippocampus (CA1) of 30-week-old R6/1 mice. Yellow arrows show nuclei with mHtt inclusions and no changes in lamin B1 levels and nuclear morphology, and blue arrows show nuclei without mHtt inclusions and changes in lamin B1 levels and nuclear morphology. Scale bar 10 μm.

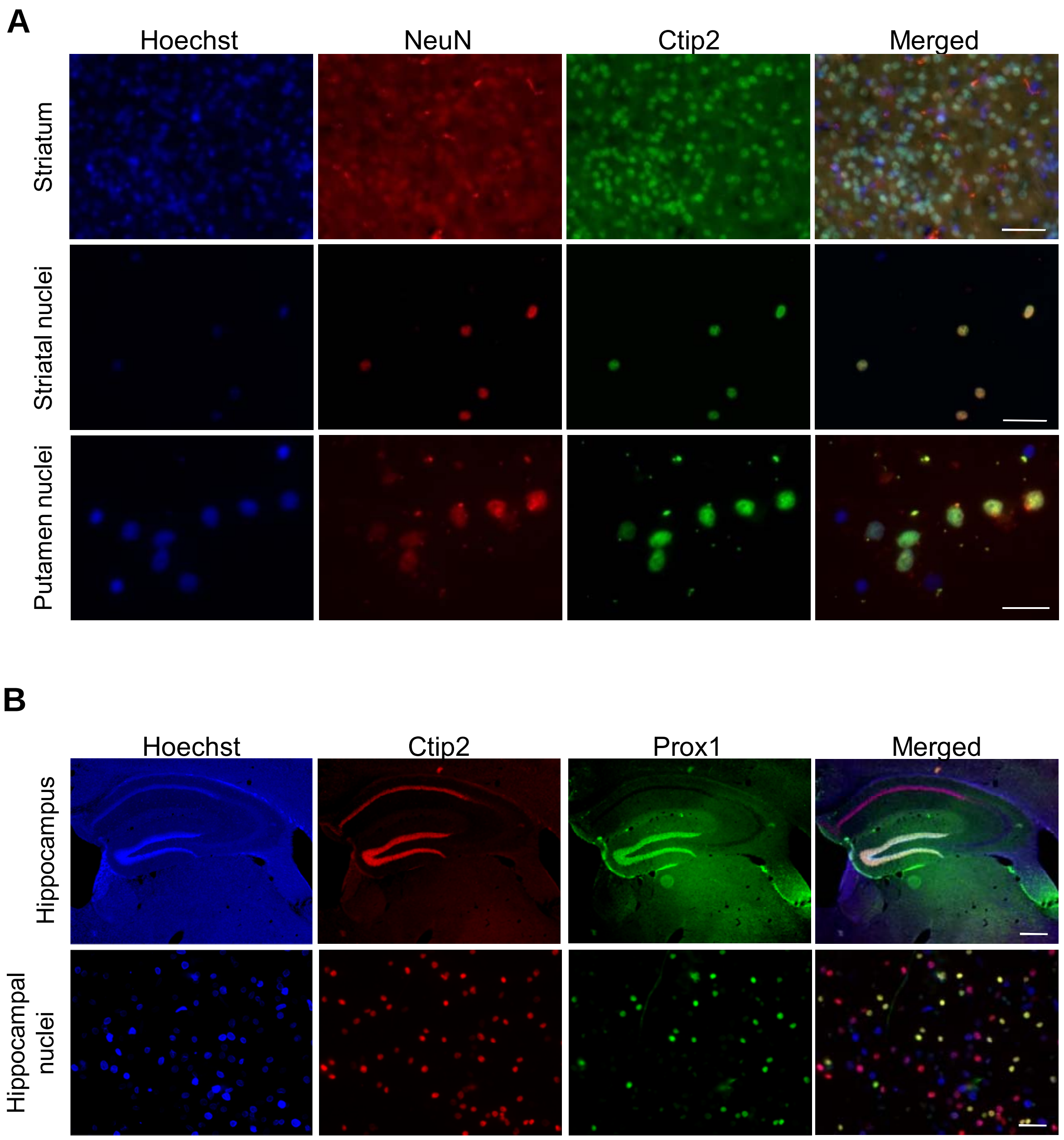

Appendix Figure S6. Nuclear markers for striatal MSNs and hippocampal CA1 and DG neurons.

A Superior panels show a striatal slice stained with Hoechst 33258 and antibodies used for nuclei selection. Images below show the correlative staining of the upper marker after the nuclear purification in R6/1 mice striatum (striatal nuclei) and in the putamen of HD patients (Putamen nuclei). The right panels represent the merge of the three other images. Classification of the nuclei can be clearly performed according to their colour being MSNs nuclei the yellow ones. Scale bars 25 μm for striatum and striatal nuclei; 10 μm for putamen nuclei.

B Superior panels show a hippocampal slice stained with Hoechst 33258 and antibodies used for nuclei selection according to their localization in the original tissue. The panels below show the correlative staining of the upper marker after the nuclear purification. The right panels represent the merge of the three other images. Classification of the nuclei can be clearly performed according to their colour (yellow for DG neurons nuclei and magenta for CA1 neurons nuclei). Scale bars 250 μm and 25 μm for hippocampus and hippocampal nuclei, respectively.

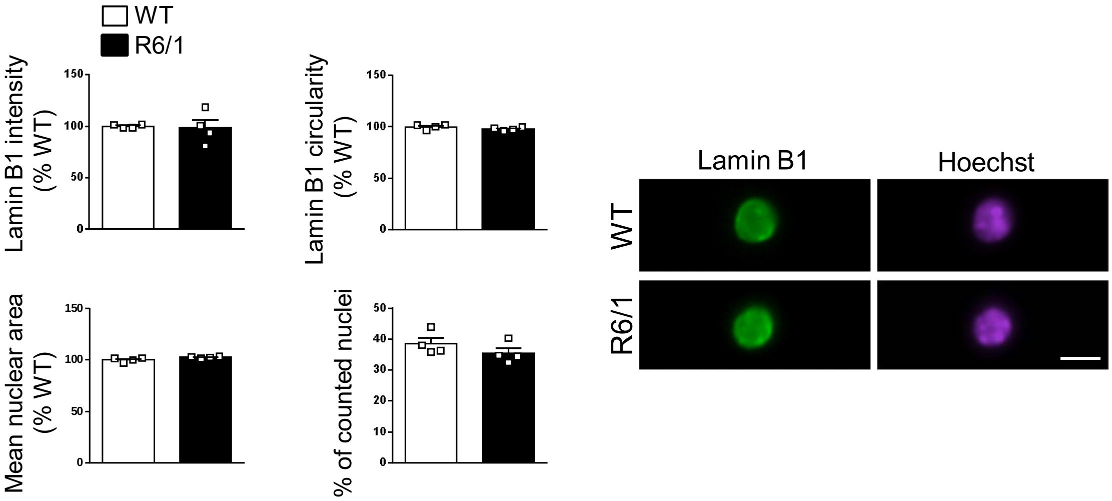

Appendix Figure S7. Lamin B1 levels and nuclear morphology are not altered in R6/1 striatal glial cells nuclei.

Graphs show the quantification by FANSI of different parameters in striatal glial nuclei (lamin B1+/Hoescht+) from 30-week-old wild-type (WT) and R6/1 mice. Each point corresponds to the value from an individual sample (*N* = 4 for each genotype). Bars represent the mean ± S.E.M Representative images are shown.

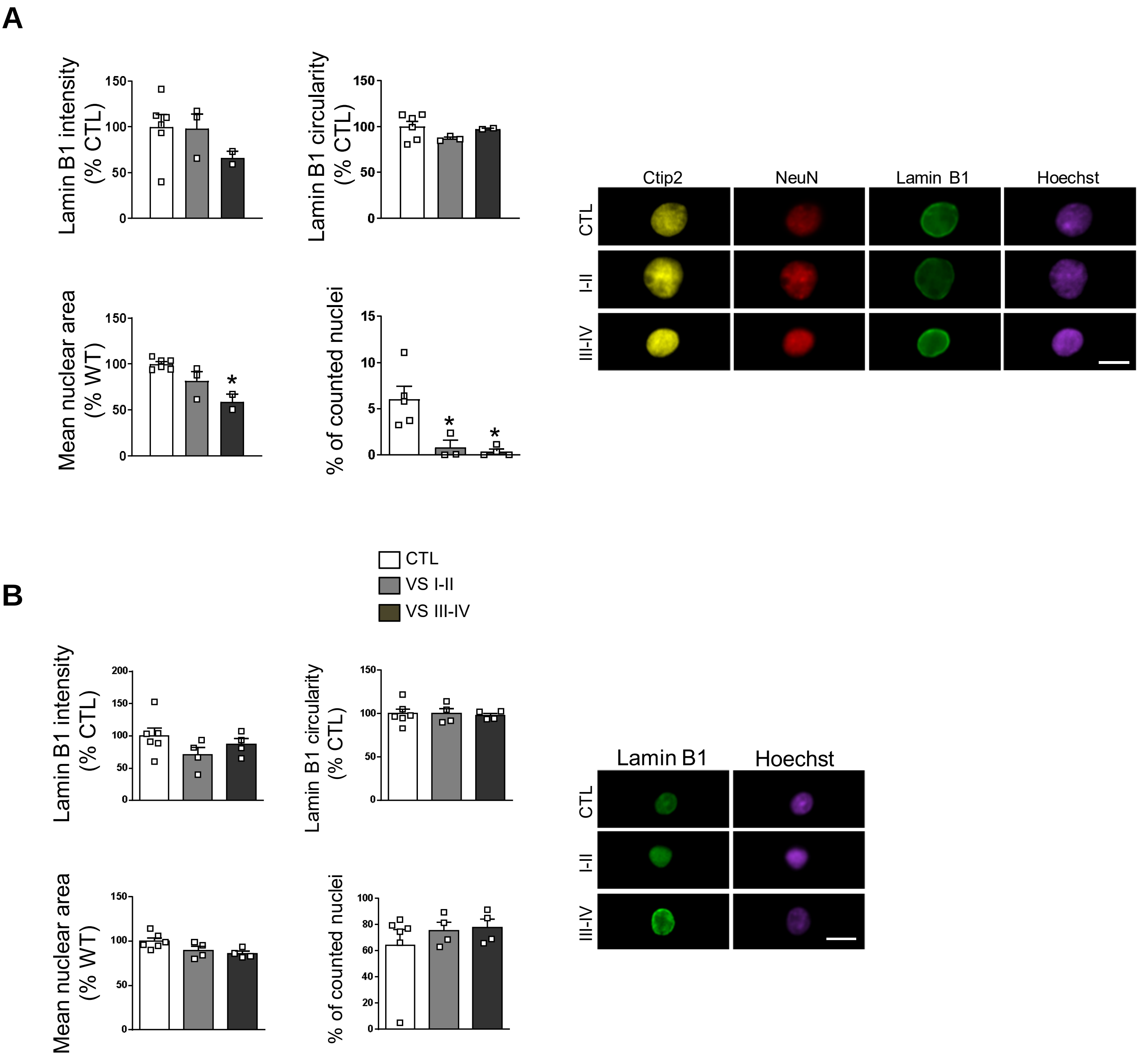

Appendix Figure S8. Analysis of MSNs and glial cells in the putamen of HD patients by FANSI.

A Graphs show the quantification of different parameters in striatal MSNs (Ctip2+/NeuN+) nuclei from HD patients at different stages of the disease (Vonsattel grades, VSI-II and III-IV). CTL *N* = 6, VS I-II *N* = 3 and VS III-IV *N* = 2. Representative images are shown.

B Graphs show the quantification of different parameters in striatal glial nuclei from HD patients at different stages of the disease (Vonsattel grades I-II and III-IV). CTL *N* = 6, VS I-II *N* = 4 and VS III-IV *N* = 4 Representative images are shown.

In graphs, each point corresponds to the value from an individual sample. Bars represent the mean ± S.E.M. *$P < 0.05$ as compared with corresponding controls (two-tailed unpaired Student's t test).

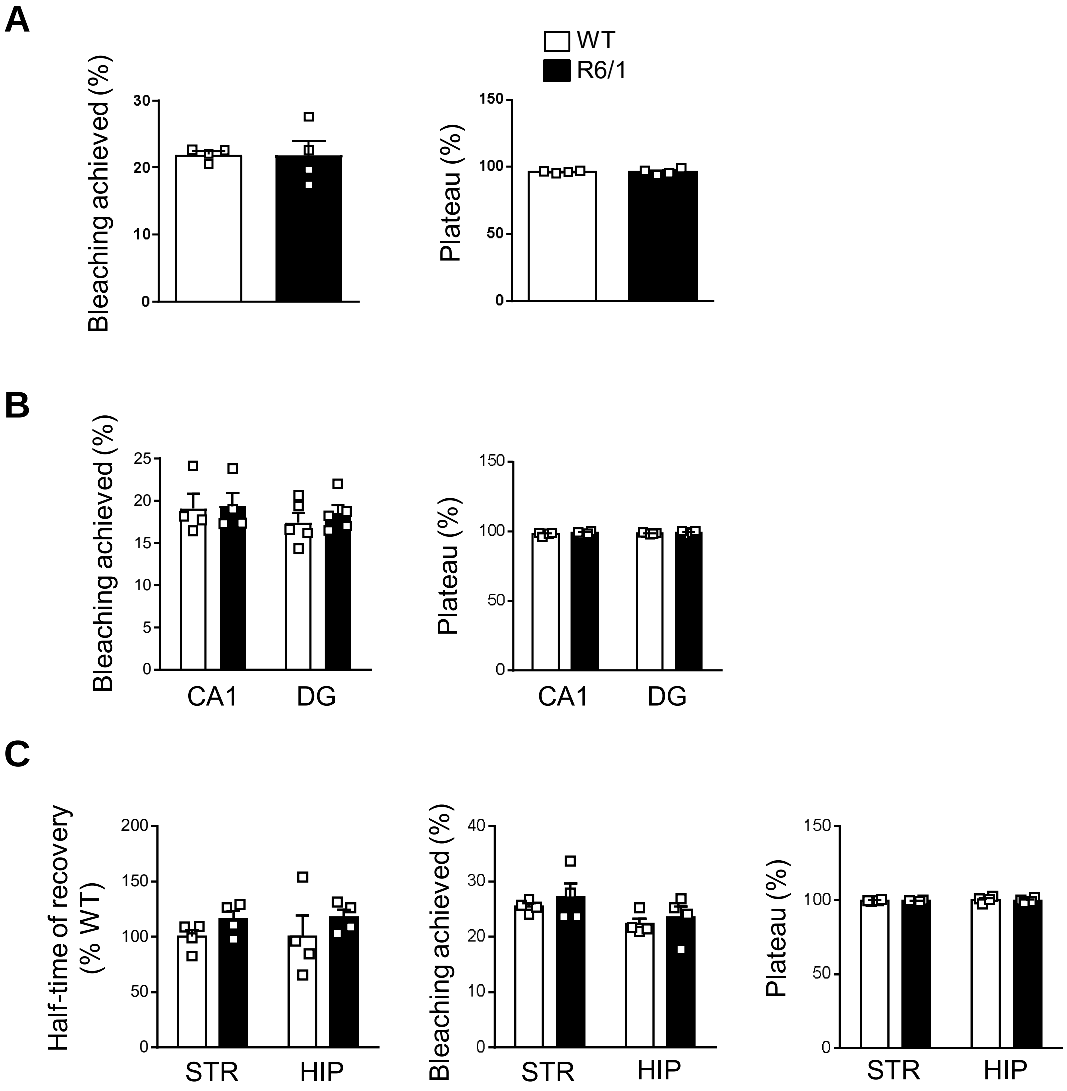

Appendix Figure S9. Dextran freely penetrates in striatal and hippocampal neurons nuclei.

A Graphs show the quantification of different parameters in MSNs nuclei from 30-week old wild-type (WT; *N* = 4) and R6/1 (*N* = 4) mice in the FRAP experiment.

B Graphs show the quantification of different parameters in hippocampal CA1 and DG nuclei from 30-week old wild-type (WT) and R6/1 mice in the FRAP experiment. CA1 *N* = 4 for both genotypes; DG *N* = 5 for both genotypes.

C FRAP experiment in the background showed no differences in any of the parameters analyzed. (WT *N* = 4; R6/1 *N* = 4).

In graphs, each point corresponds to the value from an individual sample. Bars represent the mean ± S.E.M.

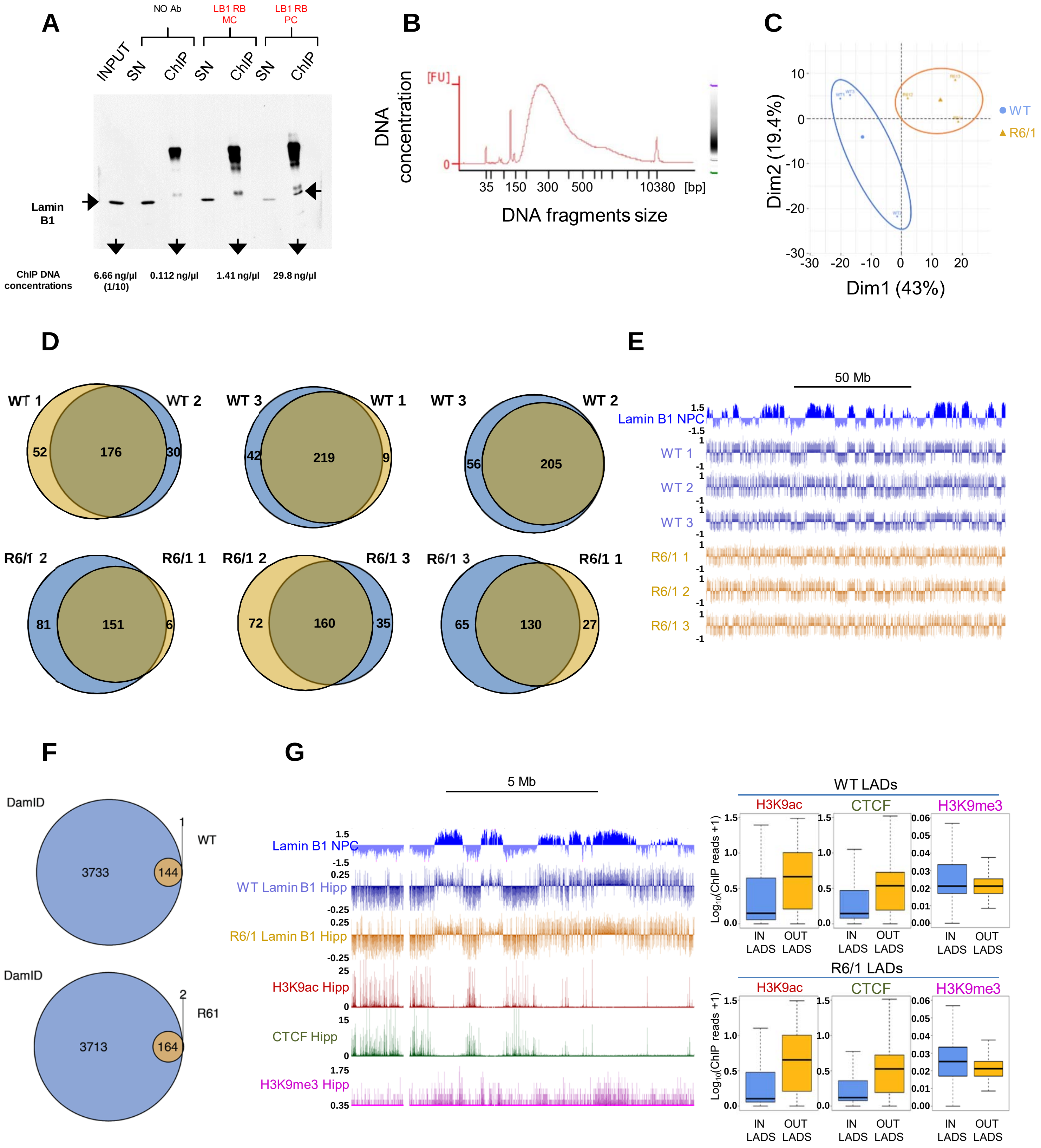
A
NO Ab
LB1 RB MC
LB1 RB PC
INPUT
SN
ChIP
SN
ChIP
SN
ChIP
Lamin B1
ChIP DNA concentrations
6.66 ng/µl (1/10)
0.112 ng/µl
1.41 ng/µl
29.8 ng/µl
B
DNA concentration
[FU]
35 150 300 500 10380 [bp]
DNA fragments size
C
Dim2 (19.4%)
Dim1 (43%)
WT
R6/1
D
WT 1
WT 2
52
176
30
WT 3
WT 1
42
219
9
WT 3
WT 2
56
205
R6/1 2
R6/1 1
81
151
6
R6/1 2
R6/1 3
72
160
35
R6/1 3
R6/1 1
65
130
27
E
50 Mb
Lamin B1 NPC
WT 1
WT 2
WT 3
R6/1 1
R6/1 2
R6/1 3
F
DamID
3733
144
1
WT
DamID
3713
164
2
R61
G
5 Mb
Lamin B1 NPC
WT Lamin B1 Hipp
R6/1 Lamin B1 Hipp
H3K9ac Hipp
CTCF Hipp
H3K9me3 Hipp
WT LADs
R6/1 LADs
H3K9ac
CTCF
H3K9me3
Log10(ChIP reads +1)
IN LADS
OUT LADS

Appendix Figure S10. Lamin B1 ChIP-seq and nuclear sub-fractionation additional data.

A Western-blot of immunoprecipitated fractions obtained from lamin B1 ChIp-seq. Lamin B1 protein levels were analysed in Input (total DNA fraction), supernatant (SN) and chromatin immunoprecipitated (ChIP) fractions in no antibody conditions (No ab), using a mouse monoclonal antibody against lamin B1 (LB1 MS MC, ab8982) and using a rabbit polyclonal antibody against lamin B1 (LB1 RB PC, ab16048). DNA concentrations of eluted DNA from each fraction is shown.

B Bioanalyzer profile of sonicated chromatin from ChIP-seq experiment.

C Principal Component Analysis (PCA) of ChIP-seq replicates using the inclusion of genes in one or more of the LADs as identified at each ChIP-seq WT (*N*=3) and R6/1 (*N*=3) experiment.

D Venn diagrams showing the number of overlapping LADs identified by EDD among the different generated replicates for WT (*N*=3) and R6/1 (*N*=3) mice datasets.

E UCSC genome browser capture of lamin B1 NPC DamID data and ChIP-seq signal (log(LB1/Input)) for all replicates generated using WT (*N*=3) and R6/1 (*N*=3) mice hippocampal tissue.

F Venn diagrams showing the number of overlapping LADs between previous DamID characterized NPCs LADs and WT (top) or R6/1 (bottom) mice hippocampus identified LADs.

G UCSC genome browser capture of lamin B1 NPC DamID data, WT and R6/1 mice hippocampus ChIP-seq signal (log(LB1/Input)) and publically available data from WT mice hippocampus of H3K9ac, CTCF and H3K9me3 ChIP- seq data (left). Boxplots showing H3K9ac, CTCF and H3K9me3 ChIP-seq data enrichment in WT (right top) and R6/1 (right bottom) mice hippocampus LADs. The bottom and top of the boxes are the first and third quartiles, and the line within represents the median. The whiskers denote the interval within 1.5 times the interquartile range (IQR) from the median.

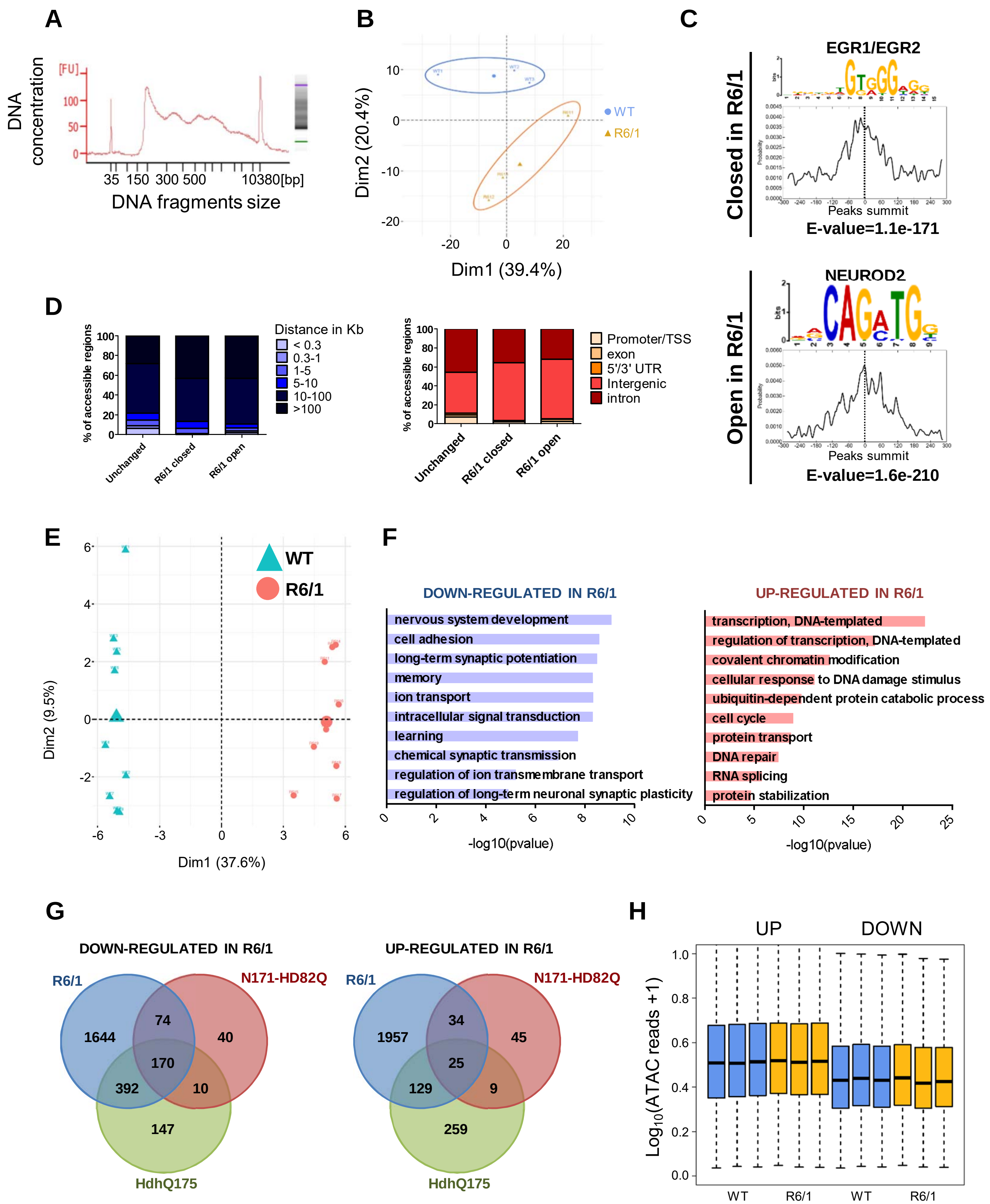

A
DNA concentration
[FU]
100
50
0
35 150 300 500 10380[bp]
DNA fragments size
B
Dim2 (20.4%)
Dim1 (39.4%)
WT
R6/1
C
EGR1/EGR2
Closed in R6/1
Peaks summit
E-value=1.1e-171
NEUROD2
Open in R6/1
Peaks summit
E-value=1.6e-210
D
% of accessible regions
Distance in Kb
< 0.3
0.3-1
1-5
5-10
10-100
>100
Unchanged
R6/1 closed
R6/1 open
Promoter/TSS
exon
5'/3' UTR
Intergenic
intron
E
WT
R6/1
Dim2 (9.5%)
Dim1 (37.6%)
F
DOWN-REGULATED IN R6/1
nervous system development
cell adhesion
long-term synaptic potentiation
memory
ion transport
intracellular signal transduction
learning
chemical synaptic transmission
regulation of ion transmembrane transport
regulation of long-term neuronal synaptic plasticity
-log10(pvalue)
UP-REGULATED IN R6/1
transcription, DNA-templated
regulation of transcription, DNA-templated
covalent chromatin modification
cellular response to DNA damage stimulus
ubiquitin-dependent protein catabolic process
cell cycle
protein transport
DNA repair
RNA splicing
protein stabilization
-log10(pvalue)
G
DOWN-REGULATED IN R6/1
R6/1
N171-HD82Q
HdhQ175
1644
74
40
170
392
10
147
UP-REGULATED IN R6/1
R6/1
N171-HD82Q
HdhQ175
1957
34
45
25
129
9
259
H
UP
DOWN
Log10(ATAC reads +1)
WT
R6/1
WT
R6/1

Appendix Figure S11. ATAC-seq and RNA-seq additional data.

A Bioanalyzer profile of transposed DNA from ATAC-seq experiments.

B Principal Component Analysis (PCA) of ATAC-seq samples with the full set of genes in the genome for WT (*N*=3) and R6/1 (*N*=3) mice data.

C Motif analysis of closed (top) and opened (bottom) chromatin regions in R6/1 mice. Associated transcription factor, motif distribution and E-value are shown for each discovered motif.

D Stacked bars graphs showing unchanged, R6/1 closed and R6/1 open chromatin regions distance to closest TSS (left) and distribution among annotated genomic categories (right).

E Principal Component Analysis (PCA) of RNA-seq data generated in WT (*N*=9) and R6/1 (*N*=9) mice hippocampus.

F Bar graphs of significant (Benjamini's adjusted *P*-value < 0.05) Biological Processes (BP) terms from DAVID for genes down- (left) and up-regulated (right) in R6/1 mice hippocampus (adjusted *P*-value <0.001). Bars represents the $-\log_{10}$ (Benjamini's adjusted *P*-value).

G Venn diagrams showing the overlap of down- (left) and up-regulated (right) genes in R6/1 mice hippocampus (adjusted *P*-value <0.001) with N171-HD82Q and HdhQ175 differentially expressed genes previously identified.

H Boxplots showing average TSS chromatin accessibility ($\log_{10}$(ATAC reads +1), N=3) for genes up or down-regulated (adjusted *P*-value<0.001, N=9) in R6/1 mice hippocampus (right). The bottom and top of the boxes are the first and third quartiles, and the line within represents the median. The whiskers denote the interval within 1.5 times the interquartile range (IQR) from the median.

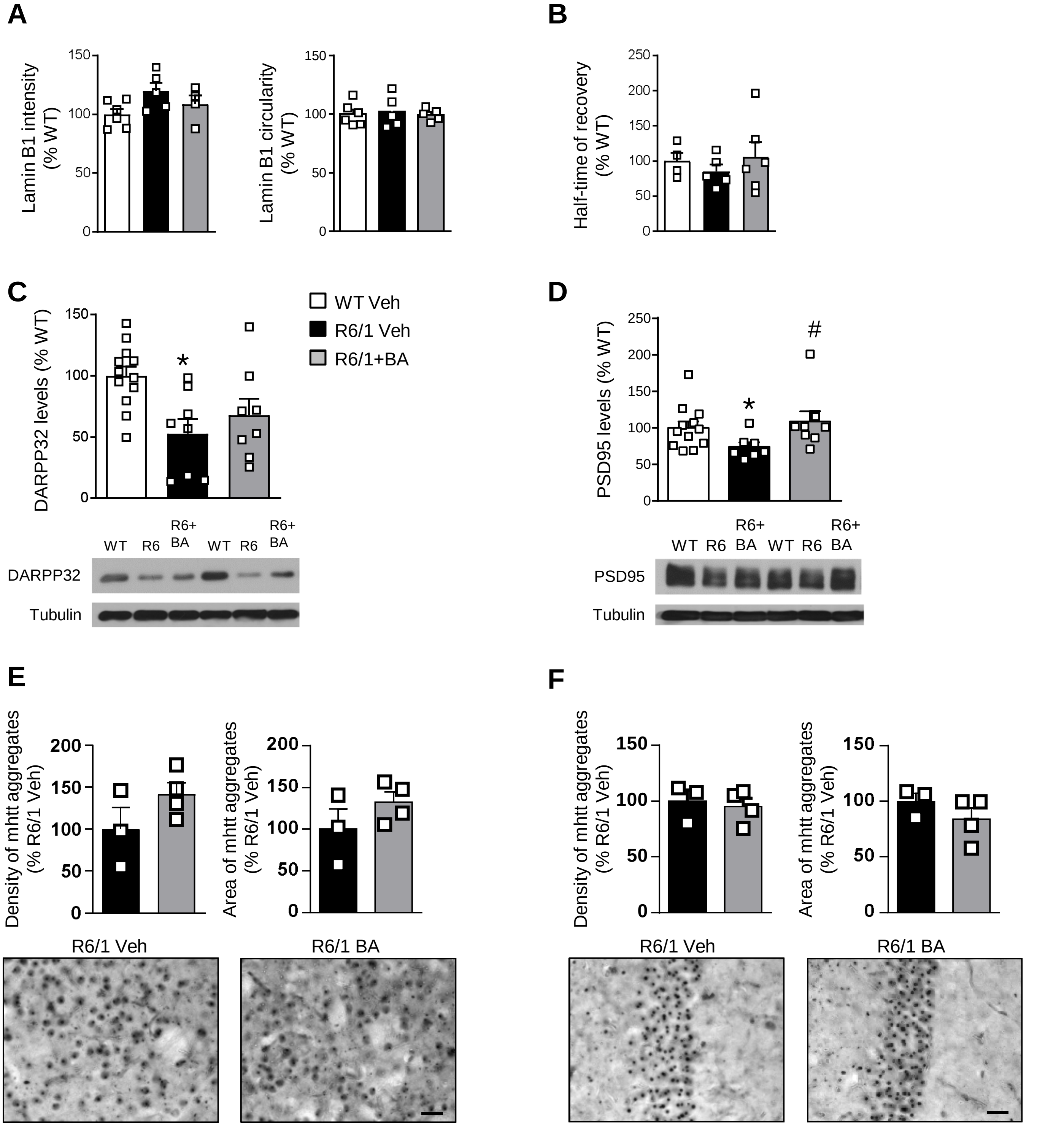

Appendix Figure S12. Effects of betulinic acid in the striatum and hippocampus at the molecular level.

A Lamin B1 intensity and circularity in the nucleus of hippocampal DG neurons (Ctip2+/Prox1+) analysed by FANSI. Graphs show the quantification in wild-type (WT) and R6/1 mice 12 weeks after treatment (Veh, vehicle; BA, betulinic acid). WT veh *N* = 6, R6/1 veh *N* = 5 and R6/1+BA *N* = 5.

B Nuclear permeability assessed by FRAP in hippocampal DG neurons nuclei from wild-type (WT) and R6/1 mice 12 weeks after treatment. WT veh N = 4, R6/1 veh N = 5 and R6/1+BA N = 6

C, D Striatal DARPP32 and hippocampal PSD95 levels in wild-type (WT) and R6/1 mice 12 weeks after treatment (veh, vehicle; BA, betulinic acid). P < 0.05 compared to vehicle-treated WT mice (one-way ANOVA followed by Bonferroni's post hoc test). WT veh *N* = 12, R6/1 veh *N* = 8 and R6/1+BA *N* = 8.

E, F Brain slices from R6/1 mice were processed for immunohistochemistry using the EM48 antibody to detect mHtt aggregates in E, the striatum and F, hippocampus. The number and area of mHtt aggregates in each group of mice were evaluated using stereological tools. Representative images are shown. Scale bar 10 μm.

In graphs, each point corresponds to the value from an individual sample. Bars represent the mean ± S.E.M.

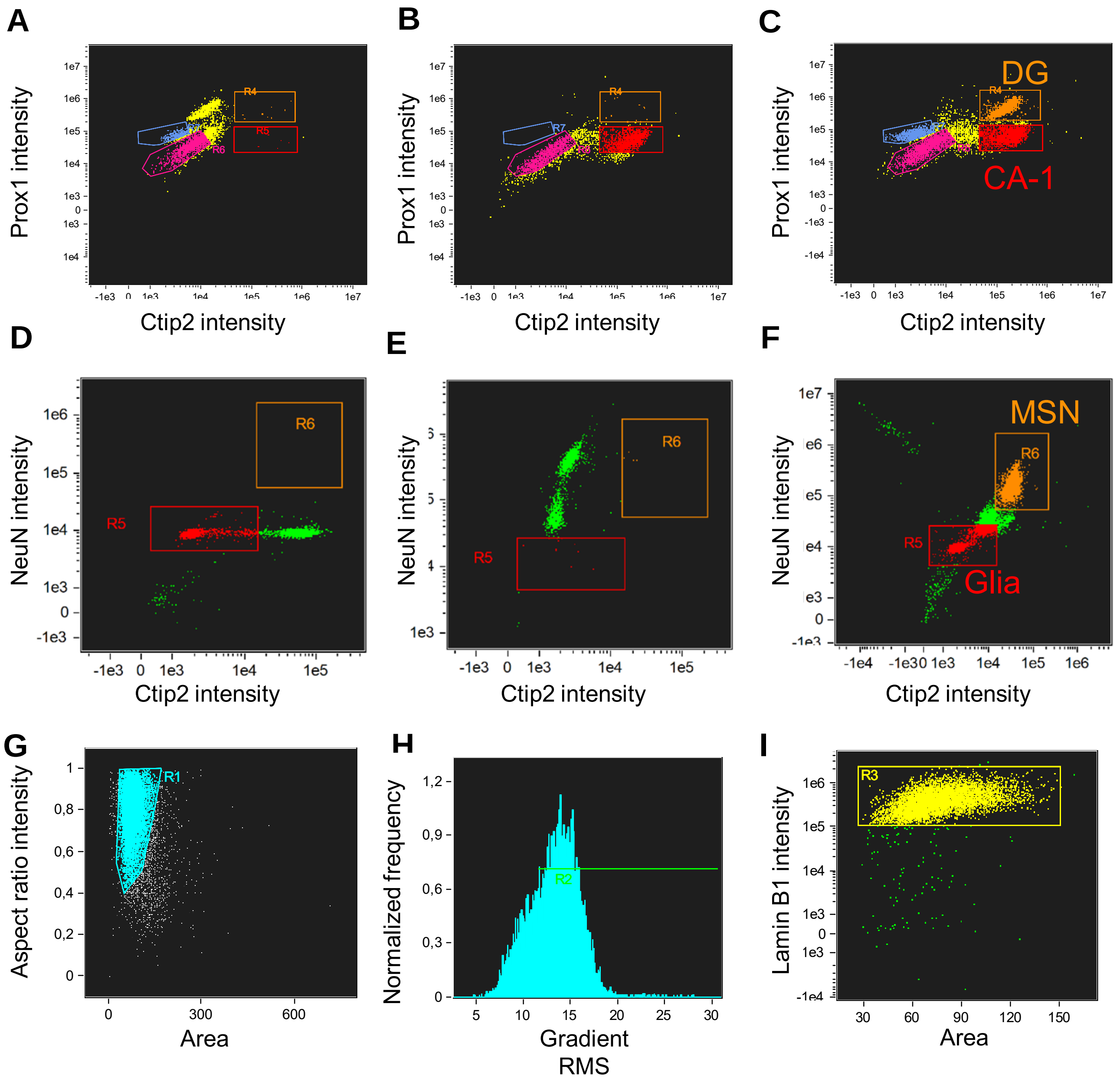

Appendix Figure S13. Selection of nuclei from neuronal populations.

A-F Identification of different populations in the hipocampus (A-C) and striatum (D-F) using FMO for the diferent antibodies (A: Ctip2; B: Prox1; D: double FMO for Ctip2 and NeuN; E: combination of single FMO for Ctip2 and NeuN). The final populations analyzed are represented (C,F).

G Representation of all the recorded events according to their area (x axis) and aspect ratio (y axis). The singlets population (individual nuclei) was selected according to the most condensed area of nuclei with an aspect ratio over 0.4 and an area of around 50-100 µm.

H Internal parameter used as a measure of focused images (gradient RMS), and only events with a gradient RMS value over 14 were accepted as correctly focused images.

I Focused events graphed according to their area and lamin B1 intensity. Only events clustered in the dense cloud were considered as real nuclei, discarding possible debris present in the sample.

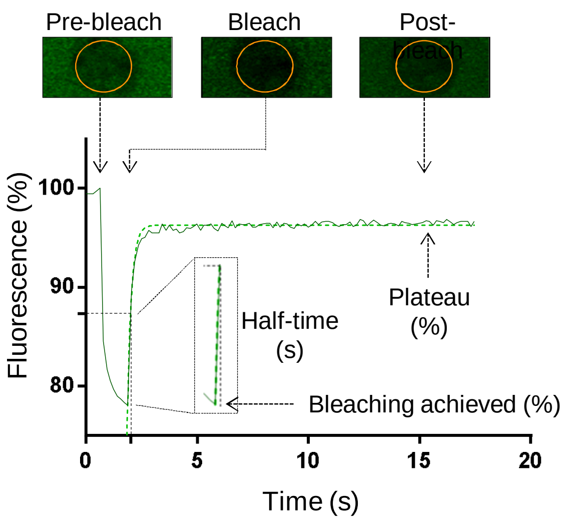

Appendix Figure S14. Representation of FRAP recovery curve with the parameters analyzed.

Representative images of pre-bleach, bleach and post-bleach phases are shown. Orange circle: ROI.

Appendix Table S1. Details on human *post-mortem* brain samples

ID	Pathological diagnosis	Gender	Age (years)	CAG repeats	PMD (hh:mm)
BK-0810	Normal	Female	81	-	23:30
BK-1570	Normal	Female	86	-	4:00
BK-1679	Normal	Female	90	-	-
BK-1774	Normal	Female	74	-	-
BK-1491	Normal	Male	83	-	13:00
BK-1563	Normal	Male	79	-	-
BK-1697	Normal	Male	78	-	6:00
BK-1733	Normal	Male	76	-	11:30
CS-1334	HD, Vonsattel grade 1	Male	73	40 +/-2	7:00
CS-1630	HD, Vonsattel grade 2	Male	76	41	6:00
CS-1638	HD, Vonsattel grade 2	Male	72	-	13:10
CS-1758	HD, Vonsattel grade 2-3	Male	68	42 +/-2	6:10
CS-1438	HD, Vonsattel grade 3	Male	85	40	5:30
CS-1120	HD, Vonsattel grade 3	Male	55	48	15:00
CS-1193	HD, Vonsattel grade 3-4	Male	55	-	7:00
CS-1294	HD, Vonsattel grade 3	Male	53	45 +/-2	7:00
BK-0801	HD, Vonsattel grade 4	Male	59	44	5:30
BK-0909	HD, Vonsattel grade 4	Male	60	43	13:05

Information about the gender, age, CAG repeat length, Vonsattel grade and *post-mortem* delay (PMD) from the 15 control individuals and 12 HD patients analyzed.

Appendix Table S2. Details on antibodies used in the study

ANTIBODY	SOURCE	IDENTIFIER	DILUTION
**WESTERN BLOT**			
Rabbit anti-Lamin B1 polyclonal antibody (human samples)	Abcam	Cat# ab16048, RRID:AB_443298	1:1000
Mouse anti-Lamin B1 monoclonal antibody (mouse samples)	Abcam	Cat# ab8982, RRID:AB_1640627	1:1000
Mouse anti-Lamin B2	Santa Cruz Biotechnology	Cat# ab18465, RRID:AB_2064130	1:1000
Rabbit anti-Lamin A/C (H-110)	Santa Cruz Biotechnology	Cat# sc-20681, RRID:AB_648154	1:1000
Mouse anti-human TATA binding protein (TBP)	Abcam	Cat# ab51841, RRID:AB_945758	1:1000
Mouse anti-PSD95 (7E3-1B8)	Thermo Fisher Scientific	Cat# MA1-046, RRID:AB_2092361	1:1000
Mouse anti-DARPP32, clone 15	BD Bioscience	Cat# 611520; RRID: AB_398980	1:1000
Mouse anti- α-tubulin	Sigma-Aldrich	Cat# T5168; RRID: AB_477579	1:50,000
Rabbit anti-PKCδ	Cell Signaling	Cat# 2058; RRID:AB_10694655	1:1000
Anti-Rabbit IgG (H+L), HRP Conjugate	Promega	Cat# W4011, RRID:AB_430833	1:2000
Anti-Mouse IgG (H+L), HRP Conjugate	Promega	Cat# W4021, RRID:AB_430834	1:2000
**IMMUNOHISTOCHEMISTRY**			
Mouse anti-Glial fibrillary acidic protein	Sigma-Aldrich	Cat# G3893, RRID:AB_477010	1.200
Rabbit anti-Lamin B1 polyclonal antibody (human samples)	Abcam	Cat# ab16048, RRID:AB_443298	1:150 (mouse sections); 1:50 (human sections)
Mouse anti-Huntingtin, clone EM48	Millipore	Cat# mab5374; RRID: AB_10055116	1:150

Mouse Anti-Olig2 Monoclonal Antibody, Unconjugated, Clone 211F1.1	Merck Millipore	Cat# MABN50, RRID: AB_10807410	1:100
Cy3-AffiniPure F(ab')2 Fragment Goat Anti-Rabbit IgG, F(ab')2 Fragment Specific	Jackson ImmunoResearch Labs	Cat# 111-166-047, RRID:AB_2338010	1:200
Cy2-AffiniPure Goat Anti-Mouse IgG (H+L)	Jackson ImmunoResearch Labs	Cat# 115-225-146, RRID:AB_2307343	1:200
**ISOLATED NUCLEI**			
Rabbit anti-Lamin B1 polyclonal antibody (human samples)	Abcam	Cat# ab16048, RRID:AB_443298	1:400 (mouse hippocampus); 1:800 (mouse striatum/human putamen)
Rat anti-Ctip-2 (25B6)	Abcam	Cat# ab18465, RRID:AB_2064130	1:400
Mouse anti-Prox1, clone 5G10	Millipore	Cat# MAB5652, RRID:AB_827462	1:200
Mouse anti-NeuN	Millipore	Cat# MAB377, RRID:AB_2298772	1:800
Alexa Fluor® 488-AffiniPure Donkey Anti-Rabbit IgG (H+L)	Jackson ImmunoResearch Labs	Cat# 711-545-152, RRID:AB_2313584	1:250
Alexa Fluor 647-AffiniPure Goat Anti-Mouse IgG (H+L)	Jackson ImmunoResearch Labs	Cat# 115-605-166, RRID:AB_2338914	1:250
Donkey F(ab')2 Anti-Rat IgG H&L (Alexa Fluor® 555) preadsorbed	Abcam	Cat# ab150150	1:250
Mouse anti-Huntingtin, clone EM48	Millipore	Cat# mab5374; RRID: AB_10055116	1:400

Appendix Table S3. Summary of statistical tests and p values of Main Figures

Figure 1A

Unpaired t test - striatum 8w	
P value	0,0201
P value summary	*
Significantly different (P < 0.05)?	Yes
One- or two-tailed P value?	Two-tailed
t, df	t=2.760, df=10
n (WT, R6/1)	6,6

Unpaired t test - striatum 12w	
P value	0,0008
P value summary	***
Significantly different (P < 0.05)?	Yes
One- or two-tailed P value?	Two-tailed
t, df	t=4.702, df=10
n (WT, R6/1)	6,6

Unpaired t test - striatum 20w	
P value	<0.0001
P value summary	****
Significantly different (P < 0.05)?	Yes
One- or two-tailed P value?	Two-tailed
t, df	t=9.168, df=10
n (WT, R6/1)	6,6

Unpaired t test - striatum 30w	
P value	0,021
P value summary	*

Significantly different (P < 0.05)?	Yes
One- or two-tailed P value?	Two-tailed
t, df	t=3.104, df=6
n (WT, R6/1)	4,4

Unpaired t test - hippocampus 8w	
P value	0,4352
P value summary	ns
Significantly different (P < 0.05)?	No
One- or two-tailed P value?	Two-tailed
t, df	t=0.8073, df=12
n (WT, R6/1)	6,6

Unpaired t test - hippocampus 12w	
P value	0,0375
P value summary	*
Significantly different? (P < 0.05)	Yes
One- or two-tailed P value?	One-tailed
t, df	t=1.949 df=12
n (WT, R6/1)	6,6

Unpaired t test - hippocampus 20w	
P value	0,0135
P value summary	*
Are means signif. different? (P < 0.05)	Yes
One- or two-tailed P value?	Two-tailed
t, df	t=3.062 df=9
n (WT, R6/1)	6,6

Unpaired t test - hippocampus 30w	
P value	<0.0001
P value summary	****

Significantly different (P < 0.05)?	Yes
One- or two-tailed P value?	Two-tailed
t, df	t=7.441, df=11
n (WT, R6/1)	6,6

Unpaired t test - cortex 8w	
P value	0,1553
P value summary	ns
Significantly different (P < 0.05)?	No
One- or two-tailed P value?	Two-tailed
t, df	t=1.593, df=7
n (WT, R6/1)	5,4

Unpaired t test - cortex 12w	
P value	0,0283
P value summary	*
Significantly different (P < 0.05)?	Yes
One- or two-tailed P value?	One-tailed
t, df	t=2.155, df=10
n (WT, R6/1)	6,6

Unpaired t test - cortex 20w	
P value	0,0378
P value summary	*
Significantly different (P < 0.05)?	Yes
One- or two-tailed P value?	One-tailed
t, df	t=2.008, df=9
n (WT, R6/1)	6,6

Unpaired t test - cortex 30w	
P value	0,0073
P value summary	**

Significantly different (P < 0.05)?	Yes
One- or two-tailed P value?	Two-tailed
t, df	t=3.449, df=9
n (WT, R6/1)	6,5

Figure 1B

Unpaired t test - striatum 8w	
P value	0,0469
P value summary	*
Significantly different (P < 0.05)?	Yes
One- or two-tailed P value?	Two-tailed
t, df	t=2.266, df=10
n (WT, R6/1)	6,6

Unpaired t test - striatum 12w	
P value	0,0299
P value summary	*
Significantly different (P < 0.05)?	Yes
One- or two-tailed P value?	Two-tailed
t, df	t=2.529, df=10
n (WT, R6/1)	6,6

Unpaired t test - striatum 20w	
P value	0,0026
P value summary	**
Significantly different (P < 0.05)?	Yes
One- or two-tailed P value?	Two-tailed
t, df	t=3.975, df=10
n (WT, R6/1)	6,6

Unpaired t test - striatum 30w	
P value	<0.0001
P value summary	****
Significantly different (P < 0.05)?	Yes
One- or two-tailed P value?	Two-tailed
t, df	t=7.975, df=8
n (WT, R6/1)	5,5

Unpaired t test - hippocampus 8w	
P value	0,4387
P value summary	ns
Significantly different (P < 0.05)?	No
One- or two-tailed P value?	Two-tailed
t, df	t=0.8011, df=12
n (WT, R6/1)	8,6

Unpaired t test - hippocampus 12w	
P value	0,7225
P value summary	ns
Significantly different (P < 0.05)?	No
One- or two-tailed P value?	Two-tailed
t, df	t=0.3643, df=11
n (WT, R6/1)	6,7

Unpaired t test - hippocampus 20w	
P value	0,0118
P value summary	*
Significantly different? (P < 0.05)	Yes
One- or two-tailed P value?	Two-tailed
t, df	t=3.244 df=8
n (WT, R6/1)	5,5

Unpaired t test - hippocampus 30w	
P value	<0.0001
P value summary	****
Significantly different (P < 0.05)?	Yes
One- or two-tailed P value?	Two-tailed
t, df	t=7.374, df=11
n (WT, R6/1)	8,5

Unpaired t test - cortex 8w	
P value	0,3273
P value summary	ns
Significantly different (P < 0.05)?	No
One- or two-tailed P value?	One-tailed
t, df	t=0.4612, df=10
n (WT, R6/1)	6,6

Unpaired t test - cortex 12w	
P value	0,0314
P value summary	*
Significantly different (P < 0.05)?	Yes
One- or two-tailed P value?	One-tailed
t, df	t=2.093, df=10
n (WT, R6/1)	6,6

Unpaired t test - cortex 20w	
P value	0,0006
P value summary	***
Significantly different (P < 0.05)?	Yes
One- or two-tailed P value?	Two-tailed
t, df	t=5.418, df=8
n (WT, R6/1)	5,5

Unpaired t test - cortex 30w	
P value	0,0087
P value summary	**
Significantly different (P < 0.05)?	Yes
One- or two-tailed P value?	Two-tailed
t, df	t=3.334, df=9
n (WT, R6/1)	6,5

Figure 4A

R6/1 vs WT - lamin B1 intensity	
Unpaired t test	
P value	0,0327
P value summary	*
Significantly different (P < 0.05)?	Yes
One- or two-tailed P value?	Two-tailed
t, df	t=2.765, df=6
n (WT, R6/1)	4,4

R6/1 vs WT - lamin B1 circularity	
Unpaired t test	
P value	0,0417
P value summary	*
Significantly different (P < 0.05)?	Yes
One- or two-tailed P value?	Two-tailed
t, df	t=2.582, df=6
n (WT, R6/1)	4,4

R6/1 vs WT - mean nuclear area	
Unpaired t test	
P value	0,1773
P value summary	ns
Significantly different (P < 0.05)?	No

One- or two-tailed P value?	Two-tailed
t, df	t=1.528, df=6
n (WT, R6/1)	4,4

R6/1 vs WT - counted nuclei	
Unpaired t test	
P value	0,0899
P value summary	ns
Significantly different (P < 0.05)?	No
One- or two-tailed P value?	Two-tailed
t, df	t=2.020, df=6
n (WT, R6/1)	4,4

Figure 4B

R6/1 vs WT - lamin B1 intensity	
Unpaired t test	
P value	0,8721
P value summary	ns
Significantly different (P < 0.05)?	No
One- or two-tailed P value?	Two-tailed
t, df	t=0.1680, df=6
n (WT, R6/1)	4,4

R6/1 vs WT - lamin B1 circularity	
Unpaired t test	
P value	0,1149
P value summary	ns
Significantly different (P < 0.05)?	No
One- or two-tailed P value?	Two-tailed
t, df	t=1.843, df=6
n (WT, R6/1)	4,4

R6/1 vs WT - mean nuclear area	
Unpaired t test	
P value	0,3846
P value summary	ns
Significantly different (P < 0.05)?	No
One- or two-tailed P value?	Two-tailed
t, df	t=0.9376, df=6
n (WT, R6/1)	4,4

R6/1 vs WT - counted nuclei	
Unpaired t test	
P value	0,6398
P value summary	ns
Significantly different (P < 0.05)?	No
One- or two-tailed P value?	Two-tailed
t, df	t=0.4926, df=6
n (WT, R6/1)	4,4

Figure 4C

mHtt+ vs mHtt- - lamin B1 intensity	
Unpaired t test	
P value	0,8422
P value summary	ns
Significantly different (P < 0.05)?	No
One- or two-tailed P value?	Two-tailed
t, df	t=0.2043, df=10
n (WT, R6/1)	6,6

mHtt+ vs mHtt- - lamin B1 circularity	
Unpaired t test	
P value	0,103
P value summary	ns
Significantly different (P < 0.05)?	No
One- or two-tailed P value?	Two-tailed
t, df	t=1.794, df=10
n (WT, R6/1)	6,6

Figure 4D

ANOVA summary - lamin B1 intensity					
F	5,959				
P value	0,0198				
P value summary	*				
Significant diff. among means (P < 0.05)?	Yes				
R square	0,5438				
Tukey's multiple comparisons test	Mean Diff.	95.00% CI of diff.	Significant?	Summary	Adjusted P Value
CTL vs. VS I-II	3,314	-20.32 to 26.95	No	ns	0,9224
CTL vs. VS III-IV	-24,03	-45.61 to -2.457	Yes	*	0,03
VS I-II vs. VS III-IV	-27,35	-52.87 to -1.818	Yes	*	0,0363
n (CTL, VS I-II, VS III-IV)	6,3,4				

ANOVA summary - lamin B1 circularity					
F	3,991				
P value	0,0532				
P value summary	ns				
Significant diff. among means (P < 0.05)?	No				
R square	0,4439				

Tukey's multiple comparisons test	Mean Diff.	95.00% CI of diff.	Significant?	Summary	Adjusted P Value
CTL vs. VS I-II	2,011	-2.702 to 6.723	No	ns	0,4963
CTL vs. VS III-IV	4,428	0.1257 to 8.730	Yes	*	0,0439
VS I-II vs. VS III-IV	2,417	-2.673 to 7.507	No	ns	0,4259
n (CTL, VS I-II, VS III-IV)	6,3,4				

Figure 4E

Two-way ANOVA - lamin B1 intensity	Ordinary				
Alpha	0,05				
Source of Variation	% of total variation	P value	P value summary	Significant?	
Interaction	22,67	0,0078	**	Yes	
Region	22,67	0,0078	**	Yes	
Genotype	27,9	0,0041	**	Yes	
Bonferroni's multiple comparisons test	Mean Diff.	95.00% CI of diff.	Significant?	Summary	Adjusted P Value
CA1:WT vs. CA1:R6/1	-33,6	-55.88 to -11.32	Yes	**	0,0028
DG:WT vs. DG:R6/1	-1,745	-24.02 to 20.53	No	ns	>0.9999
n (WT, R6/1)	4,4				

Two-way ANOVA - lamin B1 circularity	Ordinary				
Alpha	0,05				
Source of Variation	% of total variation	P value	P value summary	Significant?	
Interaction	13,09	0,0115	*	Yes	
Region	13,09	0,0115	*	Yes	

Genotype	56,1	<0.0001	****	Yes	
Bonferroni's multiple comparisons test	Mean Diff.	95.00% CI of diff.	Significant?	Summary	Adjusted P Value
CA1:WT vs. CA1:R6/1	19,76	10.12 to 29.39	Yes	***	0,0002
DG:WT vs. DG:R6/1	6,886	-2.747 to 16.52	No	ns	0,2622
n (WT, R6/1)	4,4				

Two-way ANOVA - mean nuclear area	Ordinary				
Alpha	0,05				
Source of Variation	% of total variation	P value	P value summary	Significant?	
Interaction	16,03	0,0699	ns	No	
Region	16,03	0,0699	ns	No	
Genotype	19,37	0,0492	*	Yes	
Bonferroni's multiple comparisons test	Mean Diff.	95.00% CI of diff.	Significant?	Summary	Adjusted P Value
CA1:WT vs. CA1:R6/1	0,2144	-4.628 to 5.056	No	ns	>0.9999
DG:WT vs. DG:R6/1	4,536	-0.3055 to 9.379	No	ns	0,0724
n (WT, R6/1)	4,4				

Two-way ANOVA - counted nuclei	Ordinary				
Alpha	0,05				
Source of Variation	% of total variation	P value	P value summary	Significant?	
Interaction	16,31	0,0143	*	Yes	
Region	58,94	0,0002	***	Yes	

Genotype	0,8195	0,5335	ns	No	
Bonferroni's multiple comparisons test	Mean Diff.	95.00% CI of diff.	Significant?	Summary	Adjusted P Value
CA1:WT vs. CA1:R6/1	-4,5	-13.54 to 4.540	No	ns	0,8553
DG:WT vs. DG:R6/1	7,1	-1.940 to 16.14	No	ns	0,175
n (WT, R6/1)	4,4				

Figure 5A

Unpaired t test - mean intensity	
P value	0,0432
P value summary	*
Significantly different (P < 0.05)?	Yes
One- or two-tailed P value?	Two-tailed
t, df	t=2,922, df=4
n (mApple-C1, mApple-LaminB1)	3,3

Unpaired t test - circularity	
P value	0,0051
P value summary	**
Significantly different (P < 0.05)?	Yes
One- or two-tailed P value?	Two-tailed
t, df	t=5,557, df=4
n (mApple-C1, mApple-LaminB1)	3,3

Figure 6B

Unpaired t test	
P value	0,0487
P value summary	*
Significantly different (P < 0.05)?	Yes
One- or two-tailed P value?	Two-tailed
t, df	t=2.467, df=6
n (WT, R6/1)	4,4

Figure 6C

Unpaired t test	
P value	0,0327
P value summary	*
Significantly different (P < 0.05)?	Yes
One- or two-tailed P value?	Two-tailed
t, df	t=2.763, df=6
n (WT, R6/1)	4,4

Figure 6D

Unpaired t test	
P value	0,1506
P value summary	ns
Significantly different (P < 0.05)?	No
One- or two-tailed P value?	Two-tailed
t, df	t=1.590, df=8
n (WT, R6/1)	4,4

Figure 8D

Regions closed in R6/1 vs. WT

Unpaired t test	
P value	< 0,0001
P value summary	****
Significantly different? (P < 0.05)	Yes
One- or two-tailed P value?	Two-tailed
t, df	t=10,23 df=16

Regions open in R6/1 vs. WT

Unpaired t test	
P value	< 0,0001
P value summary	****
Significantly different? (P < 0.05)	Yes
One- or two-tailed P value?	Two-tailed
t, df	t=11,12 df=16

Figure 9B

Common LADs

Unpaired t test	
P value	0,0181
P value summary	*
Significantly different? (P < 0.05)	Yes
One- or two-tailed P value?	Two-tailed
t, df	t=2,633 df=16

WT-specific LADs

Unpaired t test	
P value	0,2906
P value summary	ns
Significantly different? (P < 0.05)	No
One- or two-tailed P value?	Two-tailed
t, df	t=1,093 df=16

Figure 9C

Common LADs

Mann Whitney test	
P value	0,9
Exact or approximate P value?	Exact
P value summary	ns
Significantly different? (P < 0.05)	No
One- or two-tailed P value?	Two-tailed
Sum of ranks in column WT,R6/1	11,00 , 10,00
Mann-Whitney U	4

WT-specific LADs

Mann Whitney test	
P value	0,9
Exact or approximate P value?	Exact
P value summary	ns
Significantly different? (P < 0.05)	No
One- or two-tailed P value?	Two-tailed
Sum of ranks in column WT,R6/1	11,00 , 10,00
Mann-Whitney U	4

Figure 9E

Unchanged

Closed in R6/1

Mann Whitney test	
P value	0,8617
Exact or approximate P value?	Approximate
P value summary	ns
Significantly different? (P < 0.05)	No
One- or two-tailed P value?	Two-tailed
Sum of ranks in column WT,R6/1	1,689e+006 , 1,700e+006
Mann-Whitney U	843610

Open in R6/1

Mann Whitney test	
P value	0,0407
Exact or approximate P value?	Approximate
P value summary	*
Significantly different? (P < 0.05)	Yes
One- or two-tailed P value?	Two-tailed
Sum of ranks in column WT,R6/1	664234 , 626187
Mann-Whitney U	303381

Figure 10B

Two-way ANOVA	Ordinary				
Alpha	0,05				
Source of Variation	% of total variation	P value	P value summary	Significant?	

Interaction	25,58	<0.0001	****	Yes	
Group	2,475E-14	>0.9999	ns	No	
Object	48,52	<0.0001	****	Yes	
Bonferroni's multiple comparisons test	Predicted (LS) mean diff.	95.00% CI of diff.	Significant?	Summary	Adjusted P Value
Left Object - Right Object					
WT Veh	-31,17	-41.01 to -21.33	Yes	****	<0.0001
R6/1 Veh	-0,5792	-11.80 to 10.64	No	ns	>0.9999
R6/1 + BA	-51,63	-62.85 to -40.41	Yes	****	<0.0001
n (WT Veh, R6/1 Veh, R6/1+BA)	13,10,10				

Figure 10C

Two-way ANOVA	Ordinary				
Alpha	0,05				
Source of Variation	% of total variation	P value	P value summary	Significant?	
Interaction	25,22	<0.0001	****	Yes	
Group	3,644E-14	>0.9999	ns	No	
Object	47,35	<0.0001	****	Yes	
Bonferroni's multiple comparisons test	Predicted (LS) mean diff.	95.00% CI of diff.	Significant?	Summary	Adjusted P Value
Old Object - New Object					
WT Veh	-43,82	-54.03 to -33.60	Yes	****	<0.0001
R6/1 Veh	1,848	-9.798 to 13.49	No	ns	>0.9999
R6/1 + BA	-49,12	-60.23 to -38.02	Yes	****	<0.0001
n (WT Veh, R6/1 Veh, R6/1+BA)	13,10,11				

Figure 10D

Two-way ANOVA	Ordinary				
Alpha	0,05				
Source of Variation	% of total variation	P value	P value summary	Significant?	
Interaction	5,764	0,0312	*	Yes	
Row Factor (Day)	4,641	0,0139	*	Yes	
Column Factor (Group)	47,39	<0.0001	****	Yes	
ANOVA table	SS (Type III)	DF	MS	F (DFn, DFd)	P value
Interaction	4873	4	1218	F (4, 84) = 2.794	P=0.0312
Row Factor	3924	2	1962	F (2, 84) = 4.499	P=0.0139
Column Factor	40064	2	20032	F (2, 84) = 45.94	P<0.0001
Residual	36627	84	436		
Bonferroni's multiple comparisons test	Predicted (LS) mean diff.	95.00% CI of diff.	Significant?	Summary	Adjusted P Value
day 1					
WT Veh vs. R6/1 Veh	38,5	16.38 to 60.62	Yes	***	0,0002
WT Veh vs. R6/1 + BA	35,7	13.58 to 57.82	Yes	***	0,0005
HD Veh vs. R6/1 + BA	-2,8	-26.85 to 21.25	No	ns	>0.9999
day 2					
WT Veh vs. R6/1 Veh	29,5	7.380 to 51.62	Yes	**	0,0049
WT Veh vs. R6/1 + BA	29,7	7.580 to 51.82	Yes	**	0,0045
HD Veh vs. R6/1 + BA	0,2	-23.85 to 24.25	No	ns	>0.9999
day 3					
WT Veh vs. R6/1 Veh	70,1	47.98 to 92.22	Yes	****	<0.0001

WT Veh vs. R6/1 + BA	45,3	23.18 to 67.42	Yes	****	<0.0001
HD Veh vs. R6/1 + BA	-24,8	-48.85 to -0.7533	Yes	*	0,0409
n (WT Veh, R6/1 Veh, R6/1+BA)	13,9,9				

Figure 10E

ANOVA summary - striatum					
F	5,876				
P value	0,0098				
P value summary	**				
Significant diff. among means (P < 0.05)?	Yes				
R square	0,3701				
Bonferroni's multiple comparisons test	Mean Diff.	95.00% CI of diff.	Significant?	Summary	Adjusted P Value
WT Veh vs. R6/1 Veh	-62,51	-111.6 to -13.46	Yes	*	0,0116
WT Veh vs. R6/1 + BA	-49,9	-96.71 to -3.088	Yes	*	0,0355
n (WT Veh, R6/1 Veh, R6/1+BA)	10,6,7				

ANOVA summary - hippocampus					
F	5,743				
P value	0,0092				
P value summary	**				
Significant diff. among means (P < 0.05)?	Yes				
R square	0,3237				
Bonferroni's multiple comparisons test	Mean Diff.	95.00% CI of diff.	Significant?	Summary	Adjusted P Value
WT Veh vs. R6/1 Veh	-80,9	-138.0 to -23.76	Yes	**	0,0049
WT Veh vs. R6/1 + BA	-35,98	-95.51 to 23.55	No	ns	0,3228

n (WT Veh, R6/1 Veh, R6/1+BA)	12,8,7				

ANOVA summary - cortex					
F	3,302				
P value	0,0566				
P value summary	ns				
Significant diff. among means (P < 0.05)?	No				
R square	0,2393				
Bonferroni's multiple comparisons test	Mean Diff.	95.00% CI of diff.	Significant?	Summary	Adjusted P Value
WT Veh vs. R6/1 Veh	-33,94	-67.64 to -0.2331	Yes	*	0,0241
WT Veh vs. R6/1 + BA	-1,662	-33.77 to 30.45	No	ns	0,9018
R6/1 Veh vs. R6/1 + BA	32,28	0.4446 to 64.11	Yes	*	0,0472
n (WT Veh, R6/1 Veh, R6/1+BA)	11,6,7				

Figure 10F

Kruskal-Wallis test - lamin B1 intensity					
P value	0,0098				
Exact or approximate P value?	Exact				
P value summary	**				
Do the medians vary signif. (P < 0.05)?	Yes				
Number of groups	3				
Kruskal-Wallis statistic	7,961				
Dunn's multiple comparisons test	Mean rank diff.	Significant?	Summary	Adjusted P Value	
WT Veh vs. R6/1 Veh	-7,433	Yes	*	0,0182	
WT Veh vs. R6/1 + BA	-5,083	No	ns	0,2348	

R6/1 Veh vs. R6/1 + BA	2,35	No	ns	>0.9999	
n (WT Veh, R6/1 Veh, R6/1+BA)	6,5,4				

Kruskal-Wallis test - lamin B1 circularity				
P value	0,0036			
Exact or approximate P value?	Exact			
P value summary	**			
Do the medians vary signif. (P < 0.05)?	Yes			
Number of groups	3			
Kruskal-Wallis statistic	9,212			
Dunn's multiple comparisons test	Mean rank diff.	Significant?	Summary	Adjusted P Value
WT Veh vs. R6/1 Veh	8,4	Yes	*	0,0107
WT Veh vs. R6/1 + BA	6	No	ns	0,1122
R6/1 Veh vs. R6/1 + BA	-2,4	No	ns	>0.9999
n (WT Veh, R6/1 Veh, R6/1+BA)	6,5,5			

Figure 10G

ANOVA summary					
F	4,917				
P value	0,0276				
P value summary	*				
Significant diff. among means (P < 0.05)?	Yes				
R square	0,4504				
Bonferroni's multiple comparisons test	Mean Diff.	95.00% CI of diff.	Significant?	Summary	Adjusted P Value

WT Veh vs. R6/1 Veh	-42,94	-85.49 to -0.3815	Yes	*	0,0477
WT Veh vs. R6/1 + BA	-6,969	-47.92 to 33.98	No	ns	>0.9999

Appendix Table S4. Summary of statistical tests and p values of EV Figures

Figure EV1

Unpaired t test - striatum lamin A 8w	
P value	0,2032
P value summary	ns
Significantly different (P < 0.05)?	No
One- or two-tailed P value?	Two-tailed
t, df	t=1.362, df=10
n (WT, R6/1)	6,6

Unpaired t test - striatum lamin A 12w	
P value	0,6029
P value summary	ns
Significantly different (P < 0.05)?	No
One- or two-tailed P value?	Two-tailed
t, df	t=0.5372, df=10
n (WT, R6/1)	6,6

Unpaired t test - striatum lamin A 20w	
P value	0,2019
P value summary	ns
Significantly different (P < 0.05)?	No
One- or two-tailed P value?	Two-tailed
t, df	t=1.390, df=8
n (WT, R6/1)	5,5

Unpaired t test - striatum lamin A 30w	
P value	0,0091
P value summary	**
Significantly different (P < 0.05)?	Yes
One- or two-tailed P value?	Two-tailed
t, df	t=3.310, df=9
n (WT, R6/1)	6,5

Unpaired t test - striatum lamin C 8w	
P value	0,2774
P value summary	ns
Significantly different (P < 0.05)?	No
One- or two-tailed P value?	Two-tailed
t, df	t=1.149, df=10
n (WT, R6/1)	6,6

Unpaired t test - striatum lamin C 12w	
P value	0,8237
P value summary	ns
Significantly different (P < 0.05)?	No
One- or two-tailed P value?	Two-tailed
t, df	t=0.2287, df=10
n (WT, R6/1)	6,6

Unpaired t test - striatum lamin C 20w	
P value	0,1837
P value summary	ns
Significantly different (P < 0.05)?	No
One- or two-tailed P value?	Two-tailed
t, df	t=1.440, df=9
n (WT, R6/1)	5,6

Unpaired t test - striatum lamin C 30w	
P value	0,0075
P value summary	**
Significantly different (P < 0.05)?	Yes
One- or two-tailed P value?	Two-tailed
t, df	t=3.431, df=9
n (WT, R6/1)	6,5

Unpaired t test - hippocampus lamin A 8w	
P value	0,2891
P value summary	ns
Significantly different? (P < 0.05)	No
One- or two-tailed P value?	Two-tailed
t, df	t=1.109 df=12
n (WT, R6/1)	8,6

Unpaired t test - hippocampus lamin A 12w	
P value	0,6971
P value summary	ns
Significantly different (P < 0.05)?	No
One- or two-tailed P value?	Two-tailed
t, df	t=0.3988, df=12
n (WT, R6/1)	7,7

Unpaired t test - hippocampus lamin A 20w	
P value	0,6297
P value summary	ns
Significantly different (P < 0.05)?	No
One- or two-tailed P value?	Two-tailed
t, df	t=0.4991, df=9
n (WT, R6/1)	5,6

Unpaired t test - hippocampus lamin A 30w	
P value	0,0486
P value summary	*
Significantly different (P < 0.05)?	Yes
One- or two-tailed P value?	Two-tailed
t, df	t=2.217, df=11
n (WT, R6/1)	8,5

Unpaired t test - hippocampus lamin C 8w	
P value	0,3089
P value summary	ns
Significantly different? (P < 0.05)	No
One- or two-tailed P value?	Two-tailed
t, df	t=1.062 df=12
n (WT, R6/1)	8,6

Unpaired t test - hippocampus lamin C 12w	
P value	0,508
P value summary	ns
Significantly different (P < 0.05)?	No
One- or two-tailed P value?	Two-tailed
t, df	t=0.6822, df=12
n (WT, R6/1)	7,7

Unpaired t test - hippocampus lamin C 20w	
P value	0,2983
P value summary	ns
Significantly different (P < 0.05)?	No
One- or two-tailed P value?	Two-tailed
t, df	t=1.104, df=9
n (WT, R6/1)	5,6

Unpaired t test - hippocampus lamin C 30w	
P value	0,0197
P value summary	*
Significantly different (P < 0.05)?	Yes
One- or two-tailed P value?	Two-tailed
t, df	t=2.725, df=11
n (WT, R6/1)	8,5

Unpaired t test - cortex lamin A 8w	
P value	0,8689
P value summary	ns
Significantly different (P < 0.05)?	No
One- or two-tailed P value?	Two-tailed
t, df	t=0.1704, df=8
n (WT, R6/1)	5,5

Unpaired t test - cortex lamin A 12w	
P value	0,5946
P value summary	ns
Significantly different (P < 0.05)?	No
One- or two-tailed P value?	Two-tailed
t, df	t=0.5496, df=10
n (WT, R6/1)	6,6

Unpaired t test - cortex lamin A 20w	
P value	0,7824
P value summary	ns
Significantly different (P < 0.05)?	No
One- or two-tailed P value?	Two-tailed
t, df	t=0.2837, df=10
n (WT, R6/1)	6,6

Unpaired t test - cortex lamin A 30w	
P value	0,6546
P value summary	ns
Significantly different (P < 0.05)?	No
One- or two-tailed P value?	Two-tailed
t, df	t=0.4647, df=8
n (WT, R6/1)	6,4

Unpaired t test - cortex lamin C 8w	
P value	0,5544
P value summary	ns
Significantly different (P < 0.05)?	No
One- or two-tailed P value?	Two-tailed
t, df	t=0.6170, df=8
n (WT, R6/1)	4,6

0	
P value	0,8885
P value summary	ns
Significantly different (P < 0.05)?	No
One- or two-tailed P value?	Two-tailed
t, df	t=0.1439, df=10
n (WT, R6/1)	6,6

Unpaired t test - cortex lamin C 20w	
P value	0,4704
P value summary	ns
Significantly different (P < 0.05)?	No
One- or two-tailed P value?	Two-tailed
t, df	t=0.7502, df=10
n (WT, R6/1)	6,6

Unpaired t test - cortex lamin C 30w	
P value	0,3592
P value summary	ns
Significantly different (P < 0.05)?	No
One- or two-tailed P value?	Two-tailed
t, df	t=0.9726, df=8
n (WT, R6/1)	6,4

Figure EV2A

ANOVA summary - putamen					
F	10,87				
P value	0,0005				
P value summary	***				
Significant diff. among means (P < 0.05)?	Yes				
R square	0,497				
Bonferroni's multiple comparisons test	Mean Diff.	95.00% CI of diff.	Significant?	Summary	Adjusted P Value
CTL vs. VS I-II	-52,04	-146.9 to 42.81	No	ns	0,5074
CTL vs. VS III-IV	-139,3	-216.7 to -61.84	Yes	***	0,0004
VS I-II vs. VS III-IV	-87,24	-192.1 to 17.62	No	ns	0,1269
n (CTL, VS I-II, VS III-IV)	14,4,7				

ANOVA summary - hippocampus					
F	0,5581				
P value	0,5819				
P value summary	ns				
Significant diff. among means (P < 0.05)?	No				
R square	0,05839				
Bonferroni's multiple comparisons test	Mean Diff.	95.00% CI of diff.	Significant?	Summary	Adjusted P Value
CTL vs. VS I-II	-17,94	-100.4 to 64.53	No	ns	0,8451
CTL vs. VS III-IV	-27,24	-93.93 to 39.45	No	ns	0,5606
VS I-II vs. VS III-IV	-9,296	-93.34 to 74.75	No	ns	0,9571
n (CTL, VS I-II, VS III-IV)	9,4,8				

ANOVA summary - cortex					
F	6,024				
P value	0,0141				
P value summary	*				
Significant diff. among means (P < 0.05)?	Yes				
R square	0,481				
Bonferroni's multiple comparisons test	Mean Diff.	95.00% CI of diff.	Significant?	Summary	Adjusted P Value
CTL vs. VS I-II	-85,01	-167.3 to -2.736	Yes	*	0,042
CTL vs. VS III-IV	-84,82	-167.1 to -2.543	Yes	*	0,0425
VS I-II vs. VS III-IV	0,1928	-94.82 to 95.20	No	ns	>0.9999
n (CTL, VS I-II, VS III-IV)	8,4,4				

Figure EV2B

ANOVA summary - putamen					
F	0,7702				
P value	0,4745				
P value summary	ns				
Significant diff. among means (P < 0.05)?	No				
R square	0,06277				
Bonferroni's multiple comparisons test	Mean Diff.	95.00% CI of diff.	Significant?	Summary	Adjusted P Value
CTL vs. VS I-II	-33,06	-101.8 to 35.72	No	ns	0,6812
CTL vs. VS III-IV	-7,077	-63.02 to 48.87	No	ns	>0.9999
VS I-II vs. VS III-IV	25,98	-50.62 to 102.6	No	ns	>0.9999
n (CTL, VS I-II, VS III-IV)	15,4,7				

ANOVA summary - hippocampus					
F	0,5103				
P value	0,6138				
P value summary	ns				
Significant diff. among means (P < 0.05)?	No				
R square	0,08491				
Bonferroni's multiple comparisons test	Mean Diff.	95.00% CI of diff.	Significant?	Summary	Adjusted P Value
CTL vs. VS I-II	-38,03	-167.0 to 90.99	No	ns	>0.9999
CTL vs. VS III-IV	-39,29	-168.3 to 89.73	No	ns	>0.9999
VS I-II vs. VS III-IV	-1,262	-142.6 to 140.1	No	ns	>0.9999
n (CTL, VS I-II, VS III-IV)	6,4,4				

ANOVA summary - cortex					
F	10,69				
P value	0,0022				
P value summary	**				
Significant diff. among means (P < 0.05)?	Yes				
R square	0,6404				
Bonferroni's multiple comparisons test	Mean Diff.	95.00% CI of diff.	Significant?	Summary	Adjusted P Value
CTL vs. VS I-II	-346,4	-569.5 to -123.3	Yes	**	0,003
CTL vs. VS III-IV	-209,1	-410.9 to -7.354	Yes	*	0,0414
VS I-II vs. VS III-IV	137,3	-114.4 to 388.9	No	ns	0,466
n (CTL, VS I-II, VS III-IV)	8,3,4				

Figure EV3A

Unpaired t test	
P value	0,0155
P value summary	*
Significantly different (P < 0.05)?	Yes
One- or two-tailed P value?	Two-tailed
t, df	t=3,065, df=8
n (scramble, siRNA PKCd)	5,5

Figure EV3B

Unpaired t test	
P value	0,0429
P value summary	*
Significantly different (P < 0.05)?	Yes
One- or two-tailed P value?	Two-tailed
t, df	t=2,929, df=4
n (Scramble+mHtt, PKCd siRNA+mHtt)	3,3

Figure EV3D

Linear Regression - striatum 12w	
Best-fit values	
Slope	-0,987
Y-intercept	186,3
X-intercept	188,8
1/slope	-1,013
Std. Error	
Slope	0,2196
Y-intercept	30,11
95% Confidence Intervals	
Slope	-1.476 to -0.4976

Y-intercept	119.2 to 253.4
X-intercept	164.0 to 250.8
Goodness of Fit	
R square	0,6688
Sy.x	29,03
Is slope significantly non-zero?	
F	20,19
DFn, DFd	1, 10
P value	0,0012
Deviation from zero?	Significant
Equation	Y = -0.9870*X + 186.3
n (LB1, PKCd)	12,12

Linear Regression - striatum 30w	
Best-fit values	
Slope	-0,6046
Y-intercept	145,1
X-intercept	240
1/slope	-1,654
Std. Error	
Slope	0,2118
Y-intercept	28,6
95% Confidence Intervals	
Slope	-1.105 to -0.1037
Y-intercept	77.48 to 212.7
X-intercept	182.1 to 789.7
Goodness of Fit	
R square	0,5378
Sy.x	27,91

Is slope significantly non-zero?	
F	8,146
DFn, DFd	1, 7
P value	0,0245
Deviation from zero?	Significant
Equation	Y = -0.6046*X + 145.1
n (LB1, PKCd)	9,9

Linear Regression - hippocampus 12w	
Best-fit values	
Slope	-0,409
Y-intercept	107,3
X-intercept	262,3
1/slope	-2,445
Std. Error	
Slope	0,5188
Y-intercept	59
95% Confidence Intervals	
Slope	-1.539 to 0.7214
Y-intercept	-21.28 to 235.8
X-intercept	146.3 to +infinity
Goodness of Fit	
R square	0,04923
Sy.x	44,9
Is slope significantly non-zero?	
F	0,6214
DFn, DFd	1, 12
P value	0,4458
Deviation from zero?	Not Significant

Equation	Y = -0.4090*X + 107.3
n (LB1, PKCd)	14,14

Linear Regression - hippocampus 30w	
Best-fit values	
Slope	-0,06068
Y-intercept	95,65
X-intercept	1576
1/slope	-16,48
Std. Error	
Slope	0,0746
Y-intercept	26,42
95% Confidence Intervals	
Slope	-0.2294 to 0.1081
Y-intercept	35.88 to 155.4
X-intercept	575.8 to +infinity
Goodness of Fit	
R square	0,06849
Sy.x	52,22
Is slope significantly non-zero?	
F	0,6617
DFn, DFd	1, 9
P value	0,437
Deviation from zero?	Not Significant
Equation	Y = -0.06068*X + 95.65
n (LB1, PKCd)	11,11

Figure EV4B

Area

Unpaired t test	
P value	0,0367
P value summary	*
Are means signif. different? (P < 0.05)	Yes
One- or two-tailed P value?	Two-tailed
t, df	t=3,087 df=4

Perimeter

Unpaired t test	
P value	0,0133
P value summary	*
Are means signif. different? (P < 0.05)	Yes
One- or two-tailed P value?	Two-tailed
t, df	t=4,237 df=4

Circularity

Unpaired t test	
P value	0,0468
P value summary	*
Are means signif. different? (P < 0.05)	Yes
One- or two-tailed P value?	Two-tailed
t, df	t=2,842 df=4

Geodesic diameter

Unpaired t test	
P value	0,0086
P value summary	**
Are means signif. different? (P < 0.05)	Yes
One- or two-tailed P value?	Two-tailed
t, df	t=4,804 df=4

Tortuosity index

Unpaired t test	
P value	0,9801
P value summary	ns
Are means signif. different? (P < 0.05)	No
One- or two-tailed P value?	Two-tailed
t, df	t=0,02647 df=4

Ellipse elongation index

Unpaired t test	
P value	0,086
P value summary	ns
Are means signif. different? (P < 0.05)	No
One- or two-tailed P value?	Two-tailed
t, df	t=2,267 df=4

Convex area

Unpaired t test	
P value	0,0364
P value summary	*
Are means signif. different? (P < 0.05)	Yes
One- or two-tailed P value?	Two-tailed
t, df	t=3,095 df=4

Geodesic elongation index

Unpaired t test	
P value	0,0694
P value summary	ns
Are means signif. different? (P < 0.05)	No
One- or two-tailed P value?	Two-tailed
