# Supplementary material for: Neuron type‐specific increase in lamin B1 contributes to nuclear dysfunction in Huntington’s disease
Source: EMBO Mol Med. 2020 Dec 28;13(2):e12105. doi: 10.15252/emmm.202012105 (PMC7863407; doi:10.15252/emmm.202012105)
Supplement: Supplementary file 4 — Source Data for Figure 1 [file EMMM-13-e12105-s002.zip › Uncropped blots Fig 1.pdf]

Figure 1

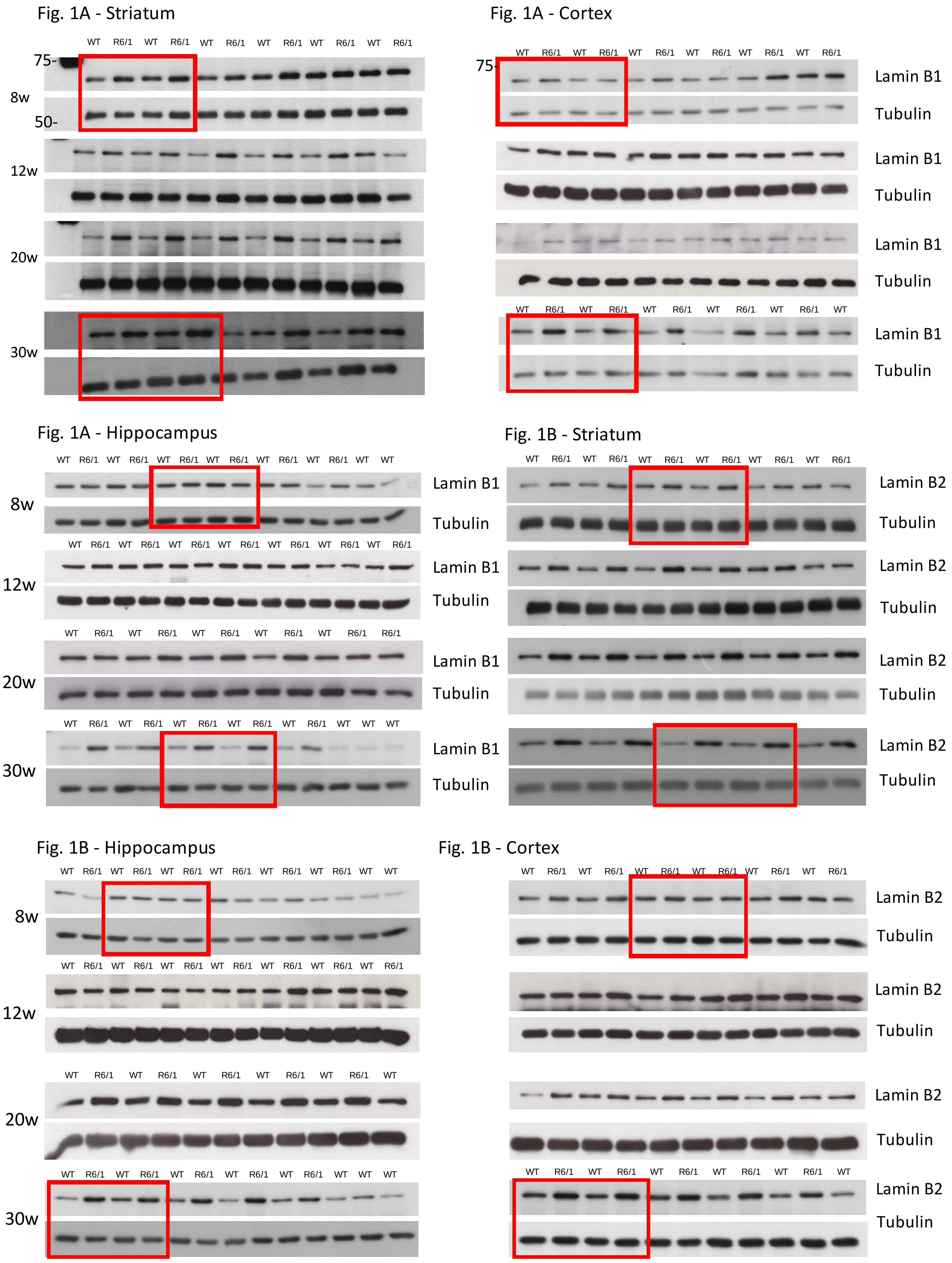

Fig. 1A - Striatum
WT R6/1 WT R6/1 WT R6/1 WT R6/1 WT R6/1 WT R6/1
75-
50-
8w
12w
20w
30w
Fig. 1A - Cortex
WT R6/1 WT R6/1 WT R6/1 WT R6/1 WT R6/1 WT R6/1
75-
Lamin B1
Tubulin
Fig. 1A - Hippocampus
Fig. 1B - Striatum
Lamin B2
Fig. 1B - Hippocampus
Fig. 1B - Cortex
