# Supplementary figures and images for: Neuron type‐specific increase in lamin B1 contributes to nuclear dysfunction in Huntington’s disease
Source: EMBO Mol Med. 2020 Dec 28;13(2):e12105. doi: 10.15252/emmm.202012105 (PMC7863407; doi:10.15252/emmm.202012105)

Figure 4D

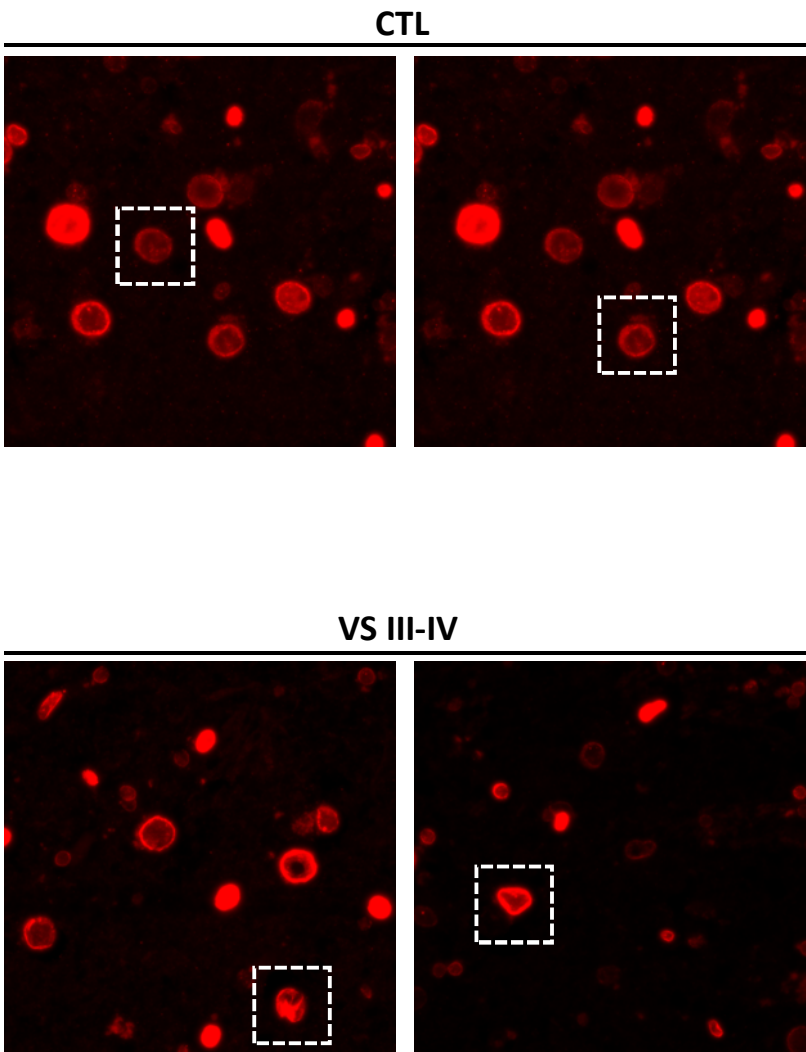

Supplement: Supplementary file 5 — Source Data for Figure 4 [file EMMM-13-e12105-s003.zip › imatges fig.4D.pdf]

mApple-C1

mApple-Lamin B1

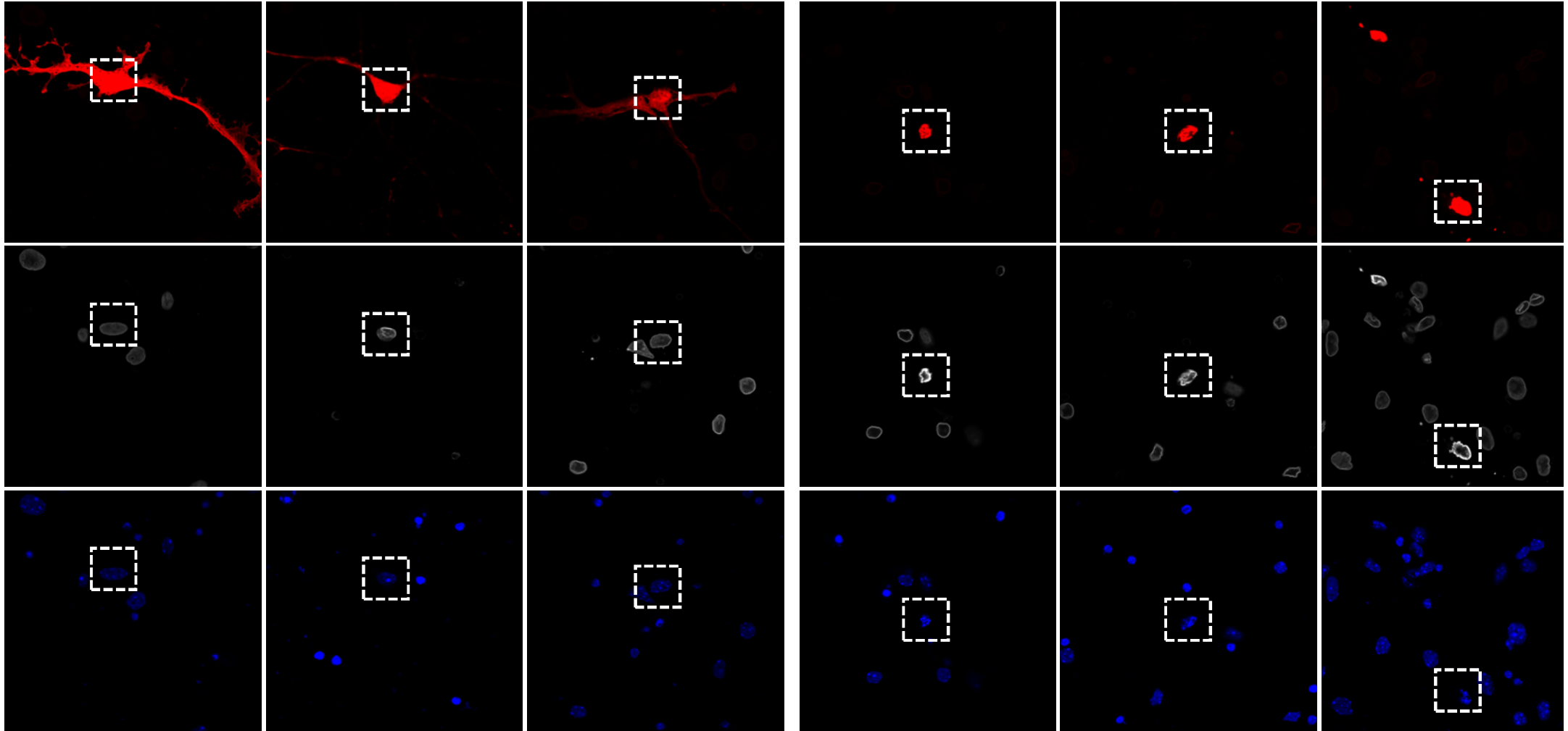

Supplement: Supplementary file 6 — Source Data for Figure 5 [file EMMM-13-e12105-s004.zip › imatges fig.5.pdf]

## Lamin B1

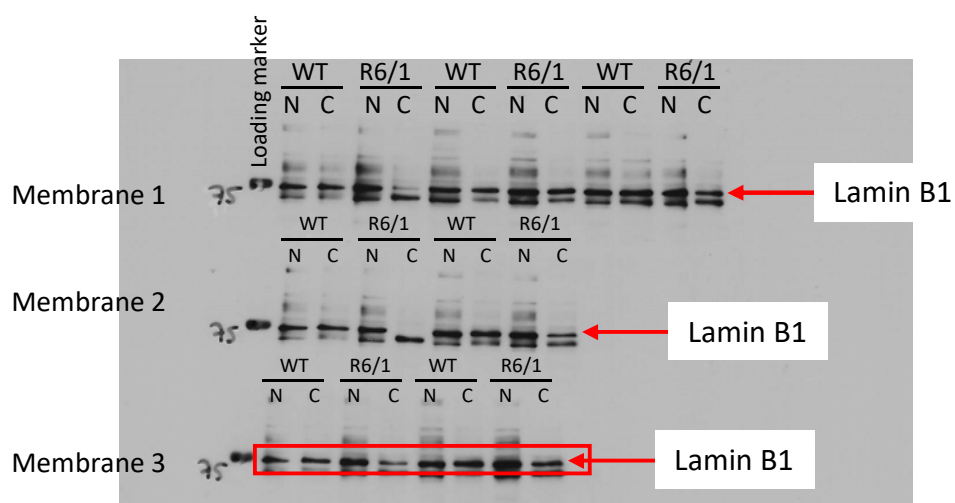

## TBP

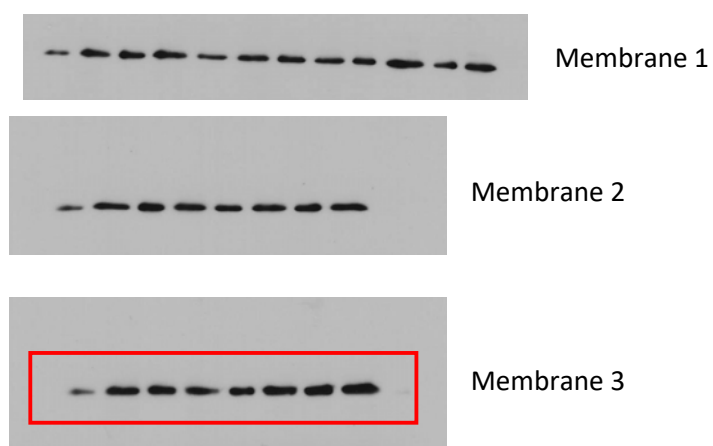

Supplement: Supplementary file 8 — Source Data for Figure 7 [file EMMM-13-e12105-s006.zip › Uncropped blots Figure 7.pdf]

Figure 10

Fig. 10E - Striatum

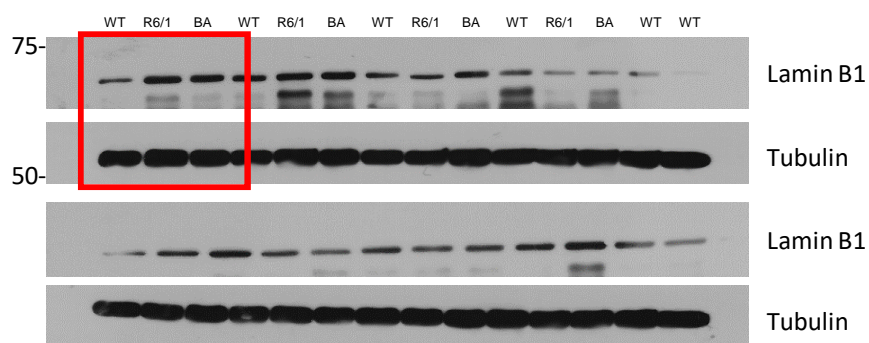

Fig. 10E - Hippocampus

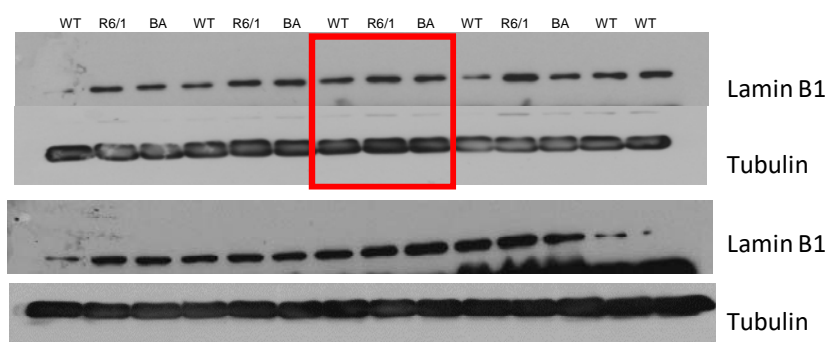

Fig. 10E - Cortex

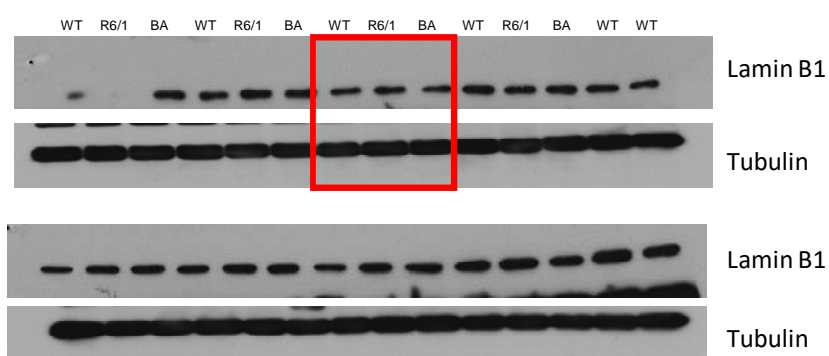

Supplement: Supplementary file 11 — Source Data for Figure 10 [file EMMM-13-e12105-s009.zip › uncropped blots Figure 10.pdf]
