# Supplementary material for: Neuron type‐specific increase in lamin B1 contributes to nuclear dysfunction in Huntington’s disease
Source: EMBO Mol Med. 2020 Dec 28;13(2):e12105. doi: 10.15252/emmm.202012105 (PMC7863407; doi:10.15252/emmm.202012105)
Supplement: Supplementary file 2 — Source Data for Expanded View [file EMMM-13-e12105-s010.zip › EMM-2020-12105-V3-Figure_EV3_Source_Data-sd.zip!/imatges fig. EV3.pdf]

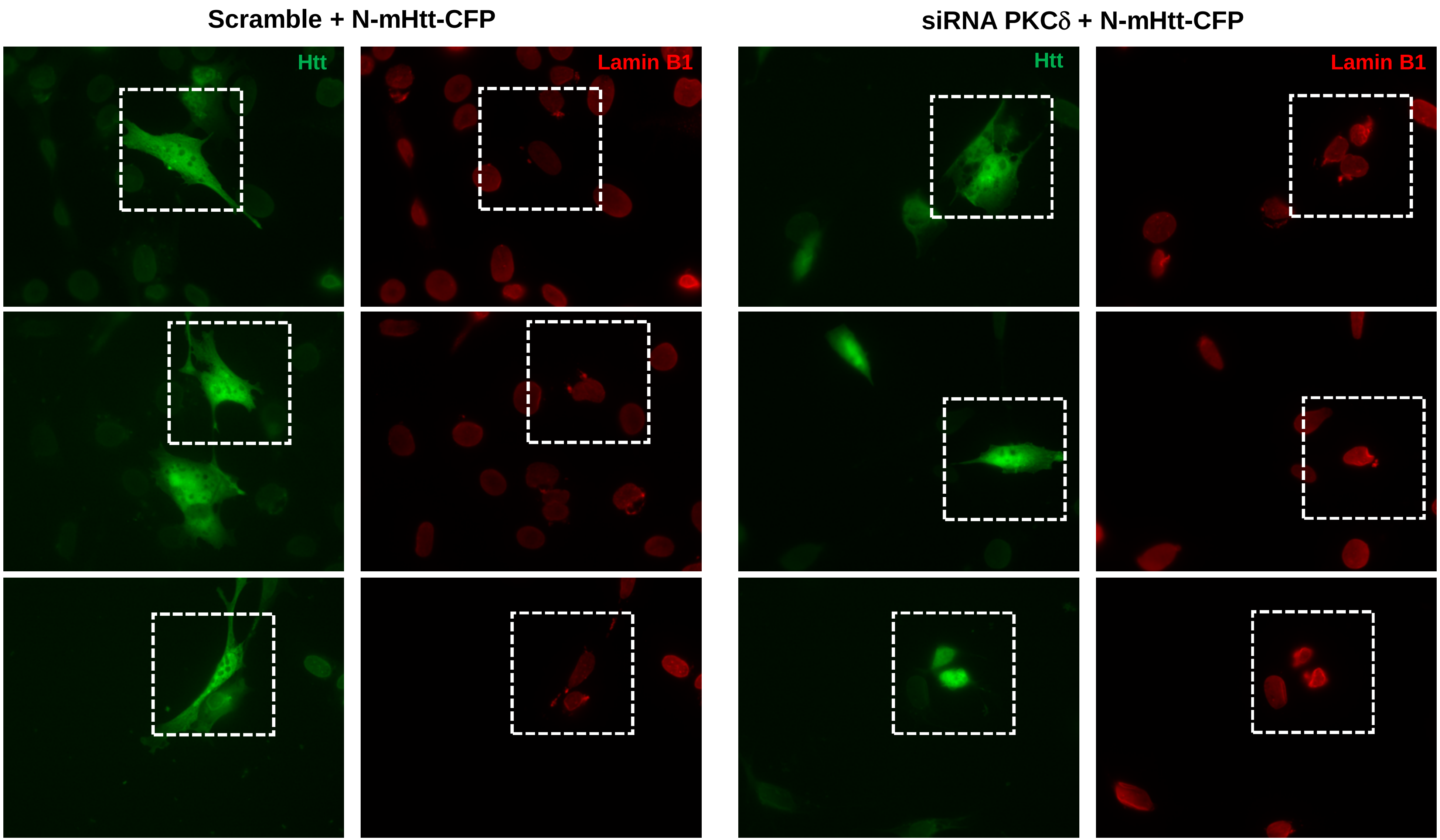
Scramble + N-mHtt-CFP
siRNA PKCδ + N-mHtt-CFP
Htt
Lamin B1
Htt
Lamin B1
