# Supplementary material for: Neuron type‐specific increase in lamin B1 contributes to nuclear dysfunction in Huntington’s disease
Source: EMBO Mol Med. 2020 Dec 28;13(2):e12105. doi: 10.15252/emmm.202012105 (PMC7863407; doi:10.15252/emmm.202012105)
Supplement: Supplementary file 2 — Source Data for Expanded View [file EMMM-13-e12105-s010.zip › EMM-2020-12105-V3-Figure_EV3_Source_Data-sd.zip!/Uncropped blots Fig. EV3.pdf]

Figure EV3A

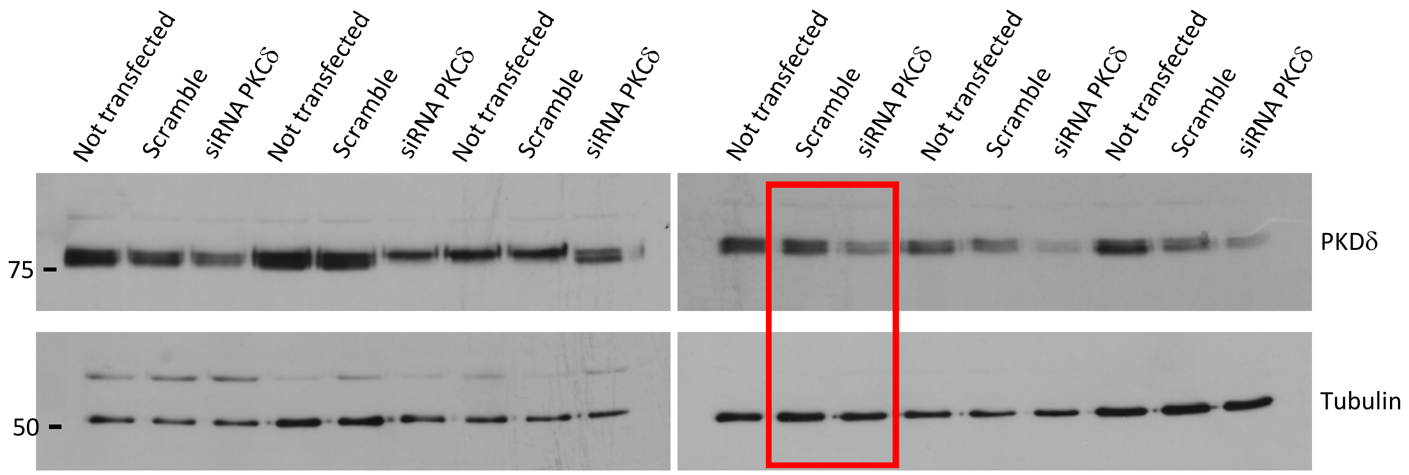
Not transfected
Scramble
siRNA PKCδ
Not transfected
Scramble
siRNA PKCδ
Not transfected
Scramble
siRNA PKCδ
Not transfected
Scramble
siRNA PKCδ
Not transfected
Scramble
siRNA PKCδ
Not transfected
Scramble
siRNA PKCδ
75
50
PKDδ
Tubulin
