# Supplementary material for: Neuron type‐specific increase in lamin B1 contributes to nuclear dysfunction in Huntington’s disease
Source: EMBO Mol Med. 2020 Dec 28;13(2):e12105. doi: 10.15252/emmm.202012105 (PMC7863407; doi:10.15252/emmm.202012105)

Figure EV2

Fig. EV2A - Putamen

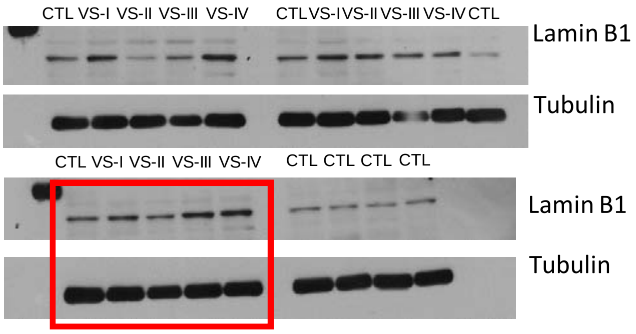

Fig. EV2A - Hippocampus

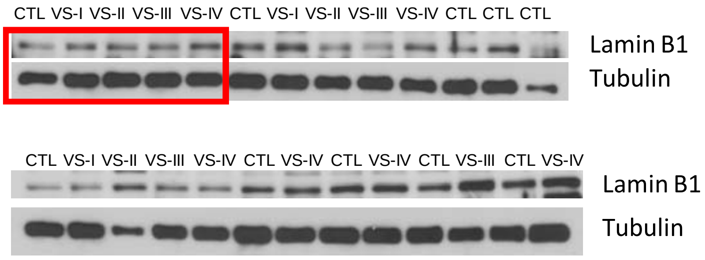

Fig. EV2A - Cortex

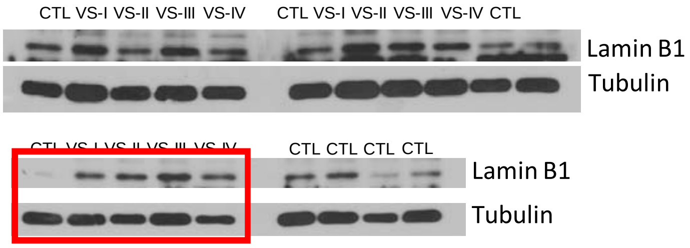

Fig. EV2B – Putamen

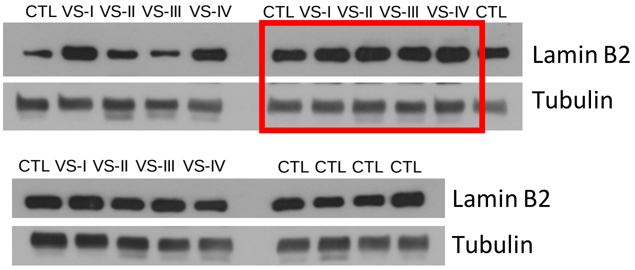

Fig. EV2B - Hippocampus

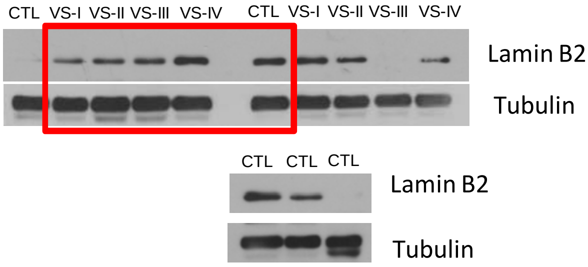

Fig. EV2B - Cortex

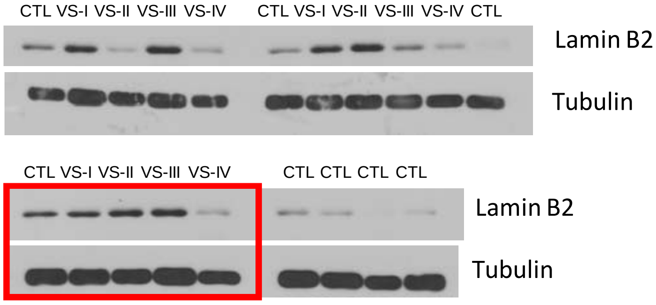

Supplement: Supplementary file 2 — Source Data for Expanded View [file EMMM-13-e12105-s010.zip › EMM-2020-12105-V3-Figure_EV2_Source_Data-sd.zip!/Uncropped blots EV2.pdf]
