# Supplementary material for: Neuron type‐specific increase in lamin B1 contributes to nuclear dysfunction in Huntington’s disease
Source: EMBO Mol Med. 2020 Dec 28;13(2):e12105. doi: 10.15252/emmm.202012105 (PMC7863407; doi:10.15252/emmm.202012105)

Figure EV1

Fig. EV1 - Striatum

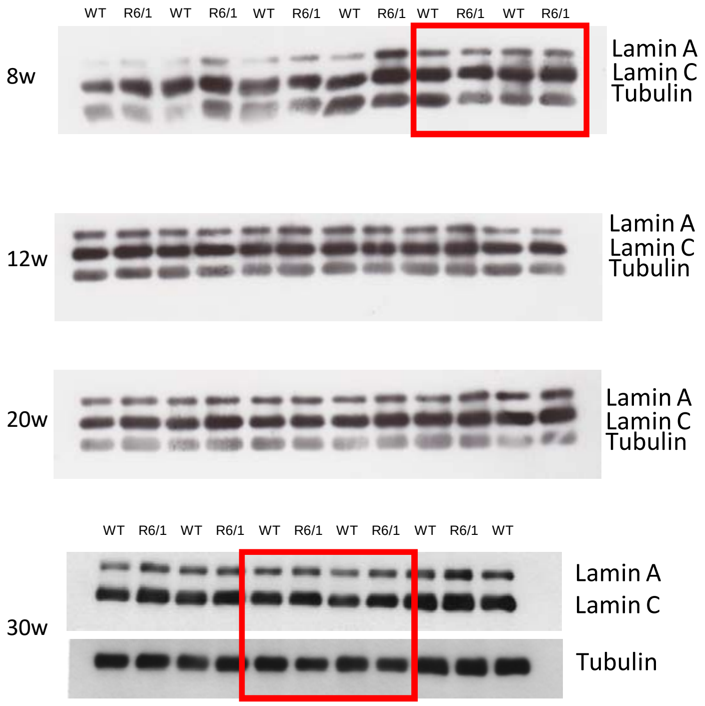

Fig. EV1 Hippocampus

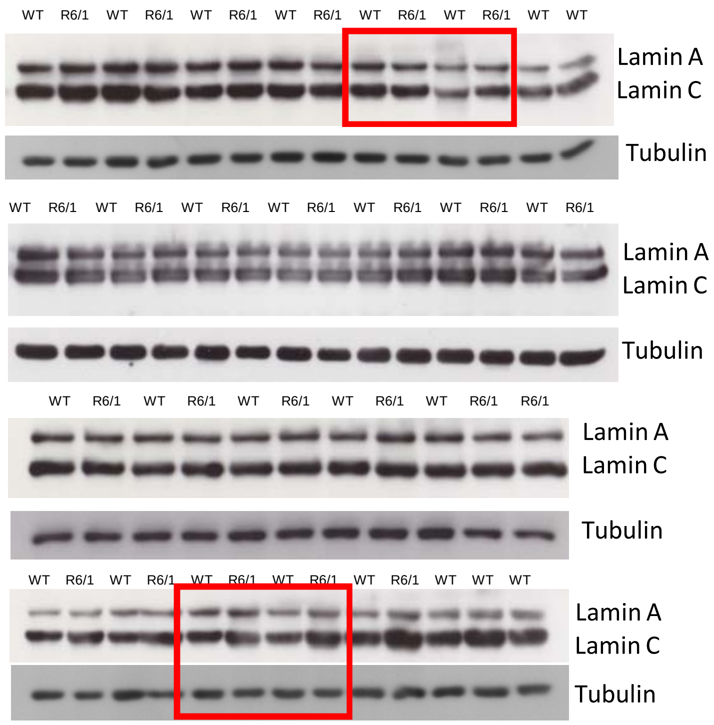

Fig. EV1 - Cortex

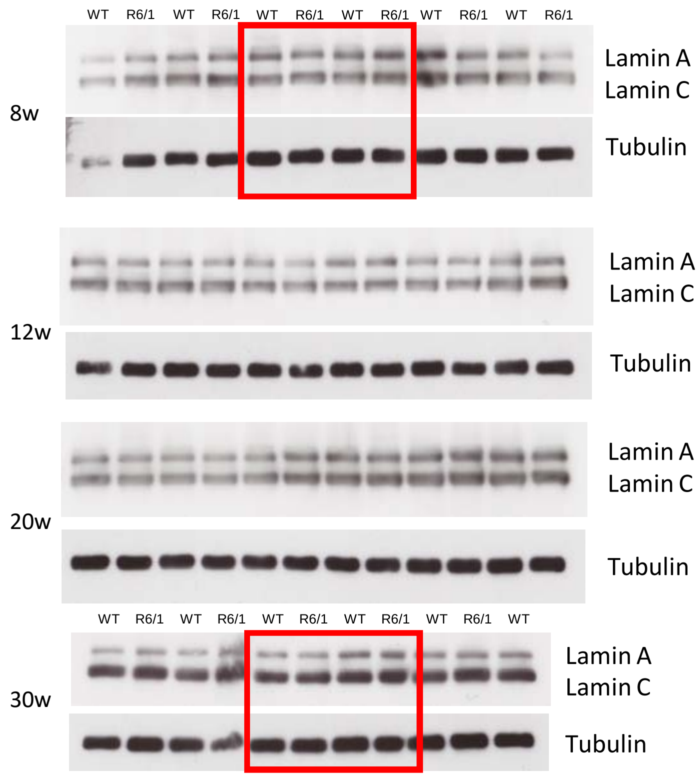

Supplement: Supplementary file 2 — Source Data for Expanded View [file EMMM-13-e12105-s010.zip › EMM-2020-12105-V3-Figure_EV1_Source_Data-sd.zip!/Uncropped blots Fig EV1.pdf]
